# Nanomaterial Integration at Liquid–Liquid Interfaces for Green Catalysis

**DOI:** 10.1002/adma.73315

**Published:** 2026-05-11

**Authors:** Bokgi Seo, Jaewon Shin, Minkyoung Jang, Kyoungho Choi, Tengfei Pang, Fangrui Zhong, Jin Woong Kim

**Affiliations:** ^1^ School of Chemical Engineering Sungkyunkwan University Suwon Republic of Korea; ^2^ College of Pharmacy Shenzhen Technology University Shenzhen China; ^3^ School of Chemisty and Chemical Engineering Huazhong University of Science and Technology Wuhan China

**Keywords:** biphasic reactions, emulsion microreactors, interfacial assembly, recoverable catalysts, Pickering emulsions

## Abstract

The assembly of functional nanomaterials at liquid–liquid interfaces offers a promising approach to address mass transfer and catalyst‐recovery limitations in conventional biphasic catalytic systems. This strategy exploits engineered colloidal particles serving dual roles as emulsion stabilizers and catalytic sites, creating platforms with high interfacial area‐to‐volume ratios. These systems can exhibit improved reaction kinetics with efficient phase separation and catalyst recyclability while potentially operating under milder conditions that reduce energy consumption and waste generation. This review analyzes recent developments in the design, synthesis, and surface engineering of interfacially active nanocatalysts. It is examined structure‐performance relationships governing catalytic efficiency and emulsion stability, assess industrial implementation challenges including scalability and economic viability, and evaluate prospects of Pickering emulsion‐based microreactor platforms as enabling technologies for sustainable chemical processes aligned with green chemistry principles and circular economy frameworks.

## Graphical abstract

Functional nanomaterials assembled at liquid–liquid interfaces create dual‐role platforms serving as emulsion stabilizers and catalytic sites, offering enhanced reaction kinetics with improved catalyst recovery and recyclability. This review examines design strategies, structure‐performance relationships, and industrial implementation prospects of Pickering emulsion‐based microreactors as enabling technologies for sustainable chemical processes.

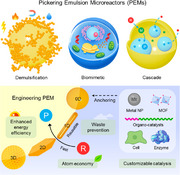

## 1 Introduction

The indispensable transition toward sustainable chemical processes became one of the defining challenges of modern society, driven by mounting concerns over environmental degradation, resource depletion, and climate change [1]. Catalysis is in the center of this transformation, with over 90% of industrial chemical processes relying on catalytic reactions to achieve the selectivity, efficiency, and atom economy demanded by contemporary manufacturing [2, 3]. However, traditional homogeneous catalytic systems, while offering high activity and selectivity, face persistent challenges in catalyst recovery, product separation, and the extensive use of organic solvents [4, 5]. These limitations become particularly acute when dealing with reactants and products of differing solubility, necessitating energy‐intensive separation processes and generating substantial waste streams [6]. Consequently, the quest for more sustainable catalytic platforms has driven researchers to explore alternative reaction environments that can reconcile catalytic performance with the principles of green chemistry [7].

**FIGURE 1:**
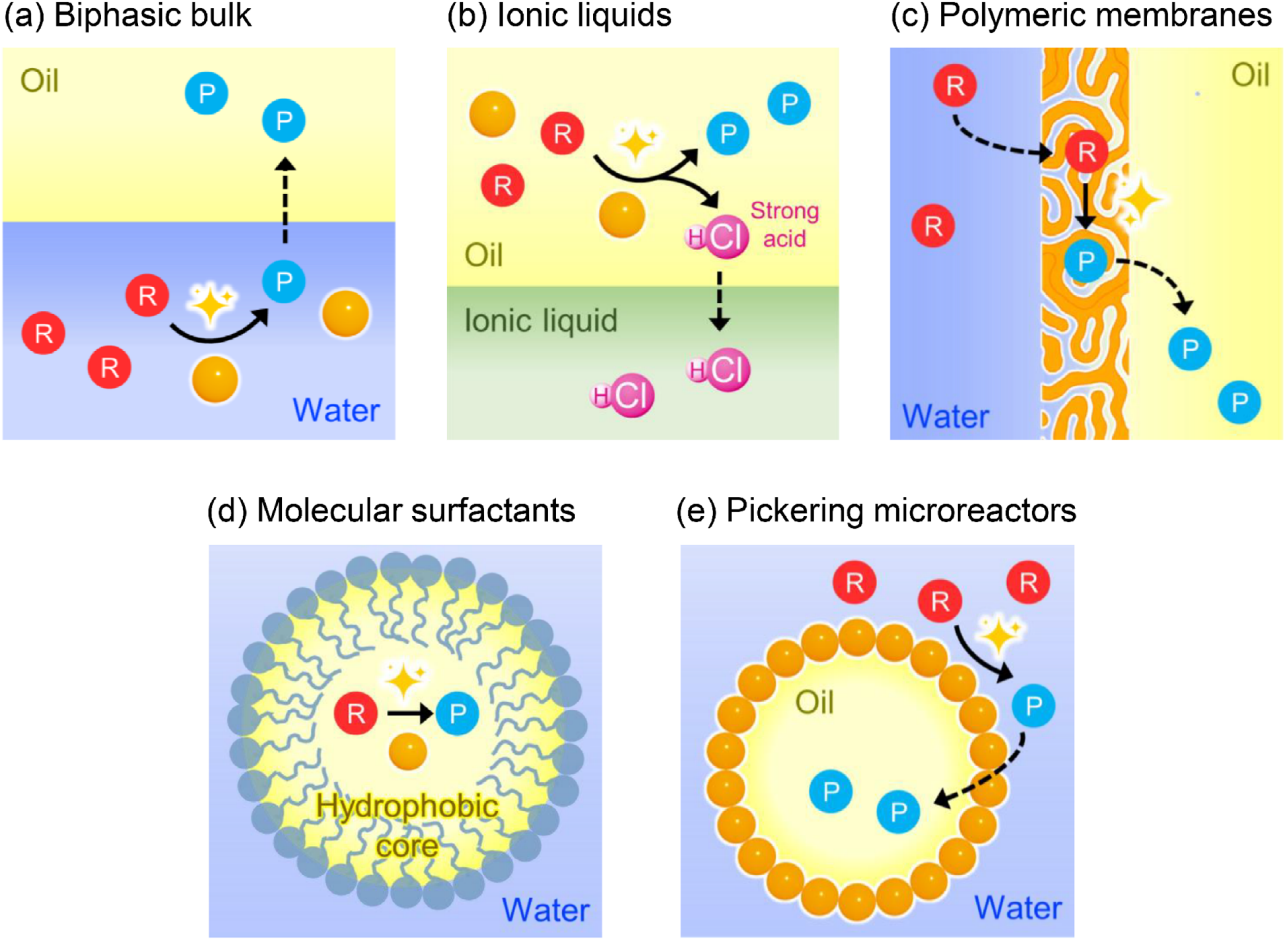
Schematic illustrations of diverse interfacial catalytic platforms. (a) Biphasic bulk, (b) ionic liquid, (c) molecular surfactant, (d) polymeric membrane, and (e) Pickering microreactors.

Reactions occurring at liquid–liquid (L–L) interfaces have been recognized as a groundbreaking approach to overcome the limitations of conventional homogeneous catalytic systems [8, 9]. Particularly for reactions where reactants and products exhibit different water solubilities, L–L systems provide an efficient platform for selective product separation and catalyst recovery [10, 11]. The most prominent reaction in biphasic bulk systems involves phase‐transfer catalyst (PTC) [12]. PTCs enable reactions between species residing in immiscible phases by transporting reactive ions or molecules across the phase boundary, thereby allowing otherwise inaccessible reactants to come into contact and undergo reaction [13]. Crown‐ethers is one of the example of them [14, 15]. Crown‐ethers are cyclic polyethers composed of repeating –CH_2–_CH_2–_O– units arranged to form a macrocyclic ring with a central cavity line by electron‐rich oxygen atoms [16]. The macrocycle adopts a conformationally flexible, toroidal structure whose interior provides a highly Lewis‐basic coordination environment capable of strongly chelating metal cations [17]. Using this cavity, crown‐ethers can deliver the cation to the organic phase for condensation reaction (Figure 1a). Ionic liquids (ILs) systems is another example that are operated in biphasic bulk systems but offer two distinct mechanistic pathways: direct catalytic action by the ILs itself, or utilization of the IL layer as a byproduct scavenger to shift equilibrium toward product formation and enhance reaction efficiency [18, 19]. The *BASF*‐developed Biphasic Acid Scavenging utilizing Ionic Liquids (BASIL) process utilized the latter approach [20]. Conventional methods required addition of tertiary amines or similar reagents for acid neutralization. The BASIL process eliminates this requirement, enabling facile separation of ILs catalyst from products while achieving high recyclability and economic viability (Figure 1b) [21]. Despite their advantages, both biphasic bulk and ILs systems exhibit a fundamental limitation: reactions occur exclusively at the interface between simply segregated aqueous and organic layers, constraining the available reaction area [22]. Interfacial catalysis are primarily governed by the mass transfer rate of chemical species between two immiscible liquid phases [23]. This recognition has driven to increase interfacial surface area and enhance mass transfer efficiency. Polymeric membrane catalysts address this challenge by positioning catalyst‐embedded membranes at the interface, where reactants encounter each other and subsequently transfer into the adjacent phase [24, 25, 26]. This configuration leverages the inherently high surface area of polymeric membranes to substantially improve reaction efficiency compared to conventional biphasic approaches (Figure 1c) [27].

Most L–L heterogenous phase catalyses are conducted in mechanically stirred tank reactors, with packed columns, agitated columns, spray columns, and static mixers serving as alternative configurations. Stirred tank reactors accommodate batch, semi‐batch, or continuous operation modes [28]. While interfacial areas depend on reactor volume and operating conditions (10–10^2^ m^2^ mL^−1^) [29]. Despite their widespread adoption, batch reactors suffer from two critical drawbacks: inhomogeneous mixing profiles and substantial energy consumption associated with continuous mechanical agitation [30, 31]. To achieve homogeneous L–L reactors with nonconsecutive stirring, researchers have pursued systems that maintain the mixing of both phases. This concept has led to the development of molecular surfactant and micellar catalyst systems [32, 33, 34]. The self‐assembly of ionic, non‐ionic, or designer surfactants in aqueous solutions enables the formation of micro‐heterogeneous environments [35]. These nanostructures act as nanoreactors, efficiently solubilizing both polar and non‐polar reactants that would otherwise be immiscible in water, thereby overcoming major mass transfer limitations [36, 37]. The enhanced efficiency stems from the unique organization at the interface. The confined space within or at the surface of micelles leads to high local concentrations of reactants and catalysts, significantly accelerating reaction rates. The confined micellar interior leads to high local concentrations, significantly accelerating various transformations including C‐H activations [38], cross‐couplings [39], oxidative coupling [40], cycloadditions [41], among others. Micellar catalysis also can be proceeded under organic solvent‐free conditions. Upon reaction completion, micelle disassembly releases the product by simple filtration and aqueous washing to remove surfactant residue [42]. The principal advantage lies in utilizing water as the primary reaction medium, substantially reducing both environmental impact and operational costs [43]. This economic and ecological benefit has driven widespread adoption in pharmaceutical companies including *Novartis*, *AbbVie*, and *Takeda* (Figure 1d). Enhanced selectivity arises from the tailored interfacial microenvironment that stabilizes specific transition states [33, 34].

An advanced approach that combines homogeneous biphasic mixing with reusuability is the Pickering emulsion microreactors (PEMs) [44]. Pickering emulsions are stabilized by solid particles which are retreievable and recylclable. This advantage of Pickering stabilizers fulfilled the requirements for the interfacial green catalysts [45]. Furthermore, PEMs dramatically enhanced the surface‐to‐volume ratio to the range of 10^4^–10^5^ m^2^ mL^−1^, which translates to superior reaction kinetics [46]. Dr. Pierre‐Gilles de Gennes introduced the concept of “Janus grains” and noted that “skin can breathe” in his 1991 Nobel lecture [47]. That metaphor described how solid particles adsorbed at interfaces create interestitial voids through which small molecules can permeate, while larger or sterically hindererd species are excluded [48]. This structural selectivity allows substrates and intermediates to reach catalytic sites efficiently without requiring solubility‐enhancing protecting groups [49]. PEMs prove particularly advantageous for phase shifting reactions involving solubility transitions between hydrophilic and hydrophobic states or hydrophobic to hydrophilic states, accommodating reversible changes in molecular polarity throughout the reaction pathway [50, 51]. Especially, PEMs are highly versatile for various reaction compared to the other interfacial catalysis by decorating the Pickering interfacial catalysts (PICs). PICs act both as the emulsion stabilizer and recyclable catalysts (Figure 1e) [45, 52]. Decorating elaborately PICs make them particularly attractive for future catalytic manufacturing [53]. PICs enable compartmentalization and spatial isolation of catalytic species, which is highly beneficial for one‐pot cascade transformations involving incompatible catalysts or reaction environments [54]. Collectively, PEMs should be studied not merely as high‐area biphasic reactors, but as promising modular platforms for process intensification, catalyst integration, and future continuous catalytic manufacturing (Table 1) [55].

**TABLE 1:** Overview of liquid–liquid biphasic interfacial catalysts.

Biphasic catalysis	Size of droplet (nm)	Reusability	Energy requirement	Catalyst/Reaction	Industrial application	Refs.
Biphasic bulk	n/a	n/a	Mid	Quaternary ammonium compounds (QAC)/phase‐transfer catalysis	Ruhr Chemie‐Rhone Poulenc (RRP)	[56]
Ionic liquid	n/a	Low; trace IL	High	Chloroaluminate IL/Alkylation	BASF's BASIL process	[20]
Polymeric membrane	n/a	Mid; pore blockage	Low	Polyethersulfone membrane‐metal/Aromatic removal	Nafion/DuraMem	[57, 58]
Molecular Surfactant	10^2^–10^5^	Low;	Low	Imidazolium salts/Suzuki coupling	Pharma‐grade micellar catalysis	[33, 34]
Pickering microreactor	10^3^–10^6^	High	Low	PIC/phase shifting catalysis	Pilot / Lab scale	[59]

* Low: Only initial agitation is required to form the emulsion.

* Mid: Gentle stirring is required during the reaction.

* High: Continuous stirring should be applied to the batch reactor throughout the reaction.

In this review, we examine the advantages of PEMs from a green catalytic perspective and survey the PEM design as governed by PIC decoration strategies. First, we elucidate how PICs with diverse dimensional morphologies can simultaneously stabilize the liquid–liquid interface while exploiting their unique structural features to facilitate catalytic transformations. Subsequently, we discuss surface‐functionalized strategies including organic, inorganic, and chemo‐bio catalytic variants. This is followed by an examination of stimuli‐responsive PEMs engineered for facile catalyst recovery and recycling. Finally, we discuss classification of advanced PEM which can procced the cascade reaction including Janus particle, Pickering interfacial biocatalyst, continous flow microreactor (CFM), double emulsion templated microreactor, and artificial biomimetic enzymes.

## 2 Interfacial Catalysis in Green Chemistry Framework

**FIGURE 2:**
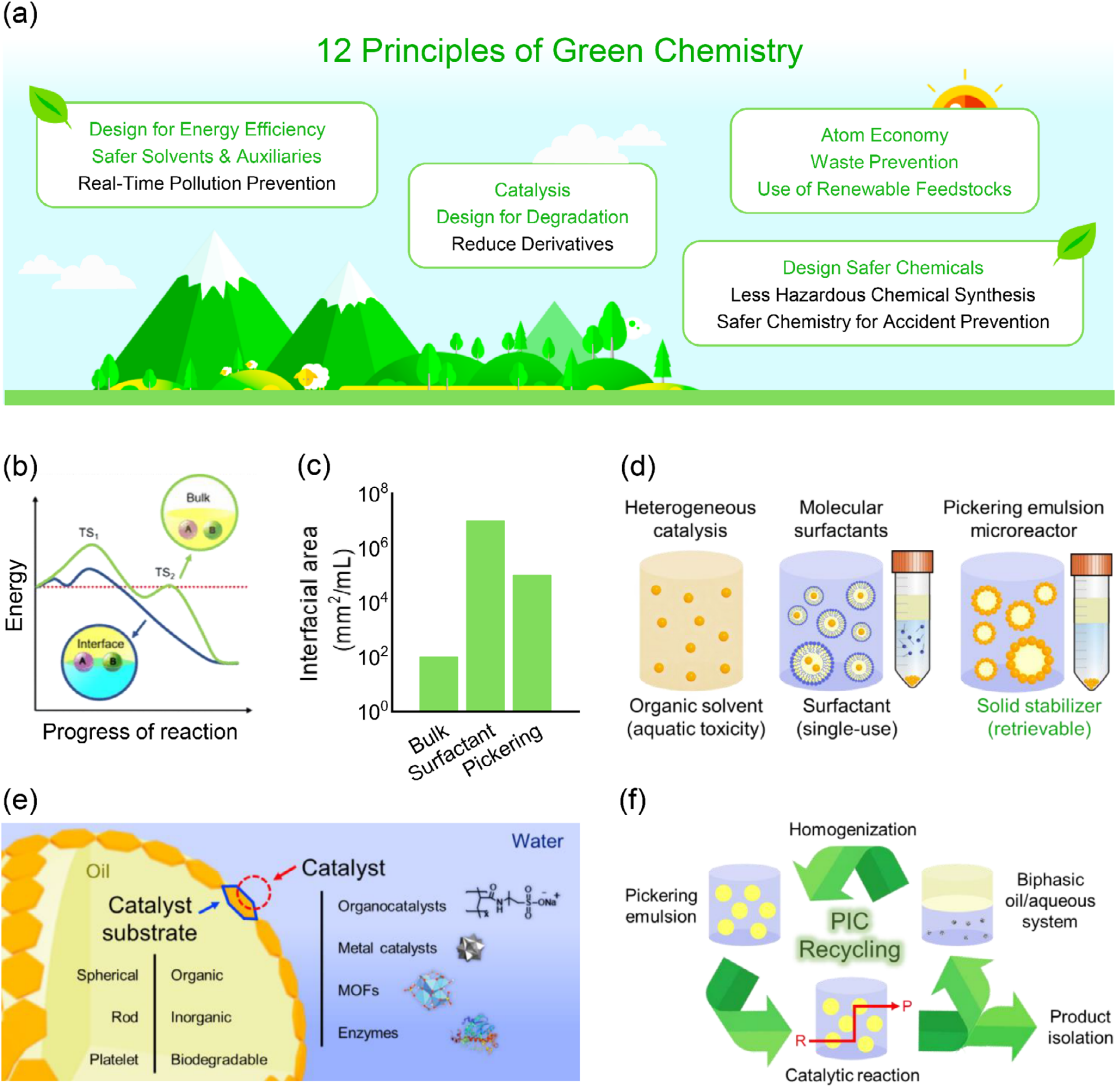
Alignment of PEMs with green chemistry. (a) The 12 principles of green chemistry. Reproduced with permission [66]. Copyright 2000, Oxford Academic. (b) Theoretical comparison of activation energy at the biphasic interfaces and in the homogenous system and (c) comparison of interfacial area to volume ratio in various biphasic catalytic systems. Reproduced with permission [60]. Copyright 2024, American Chemical Society. (d) Sustainability advantages of PEMs over conventional biphasic catalytic systems, (e) versatile designs of interfacial catalysts, and (f) recycling pathways.

Conventional catalytic processes struggle with excessive energy consumption, heavy solvent use, poor catalyst recovery, and dependence on finite fossil feedstocks [60]. These limitations directly conflict with global carbon regulations and environmental standards that increasingly penalize carbon‐intensive operations [61, 62]. In response, Anastas and Warner developed the 12 principles of green chemistry as a framework for designing sustainable processes, now widely across academia and the chemical industry (Figure 2a) [63, 64]. PEMs offers a compelling solution by integrating multiple green chemistry principles into their fundamental architecture, fulfilling both the technical demands of next‐generation catalysis and the regulatory pressures of a carbon‐constrained future [65].

The microcompartment structure of these systems fundamentally reshapes reaction thermodynamics in ways unattainable in bulk media. Enhanced equilibrium constant (*K*_eq_) stem from a unique reaction‐adsorption phenomenon: reactants within emulsion microdroplets adsorbs to boundaries at lower energy states, reducing the Gibbs free energies (*ΔG^0^*) of transition states inversely proportional to the droplet radius (Figure 2b) [46, 67, 68]. This process intensification enables reactions under significantly milder conditions, directly aligning with “*design for energy efficiency*.”

Bulk reactions are transformed into countless microscopic reactors, each droplet providing confined reaction space with extraordinary surface area (Figure 2c) [46]. The total L–L interfacial area (*A*) in an emulsion can be estimated from the dispersed phase volume fraction (*φ*) and the Sauter mean droplet diameter (*d_32_*) as:

(1) $$A=\frac{6\varphi }{d_{32}}\;$$

where droplet miniaturization increases the total accessible liquid–liquid interfacial area at a fixed disperse phase fraction [69]. The characteristic diffusion time (τ) for molecular transport within a spherical emulsion droplet with the droplet radius (*r_0_*), the molecular diffusion coefficient (*D*), and the interfacial exchange rate constant (α) can be expressed as:

(2) $$\bar{\tau }=\frac{r_{0}^{2}}{15D}+\frac{r_{0}}{3\alpha }$$

Accordingly, the characteristic transport time decreases with the square of the droplet size, demonstrating accelerated mass transfer in compartmentalized emulsion microreactors [70, 71]. Therefore, smaller droplets simultaneously maximize interfacial area and minimize transport resistance, allowing concentrated catalysts and reactants to operate across drastically shortened mass‐transfer pathways. Such acceleration of reaction rates and conversion efficiency embodies the principle of “*reduced derivatives*.”. This distincitve interfacial archtecture excels at implementing “*use of renewable feedstocks*” and “*safer solvents and auxiliaries*.” Catalyst‐functionalized particles jam irreversibly at L–L boundaries, eliminating surfactants and toxic solvents while creating highly active catalytic surfaces [72]. Hazardous solvent consumption and auxiliary chemical use are markedly reduced, permitting water‐rich or benign solvent sytems (Figure 2d).

PEMs offer remarkable versatility in “*catalysis*.” Diverse catalytic moieties, ranging from metal nanoparticles and organocatalytic moieties to polymer‐bound enzymes, can be anchored onto the stabilizing particles at the interface, enabling highly customizable and reaction‐specific platforms (Figure 2e) [45, 73, 74]. When these stabilizers incorporate biodegradable or bio‐based materials, they fulfill “*design for degradation*,” multiplying sustainability benefits [75]. Effortless catalyst recycling represents another key advantage. Post‐reaction, simple centrifugation or controlled demulsification cleanly separates phases without downstream purification steps commonly needed in bulk systems [76, 77]. The recovered catalyst particles immediately reassemble into fresh emulsions, regenerating the microreactor architecture for successive cycles (Figure 2f) [78]. This circular workflow exemplifies both “*waste prevention*” and “*atom economy*,” maximizing material efficiency across repeated operations. Collectively, these systems unify reaction intensification, safer chemistry, renewable materials, and circular catalyst use within a single interfacial platform. This interfacial engineering translates the abstract principles of green chemistry into practical, high‐performance catalytic systems ready for industrial depolyment.

## 3 Engineering Colloidal Morphology for Spatially Controlled PEM Interfaces

**FIGURE 3:**
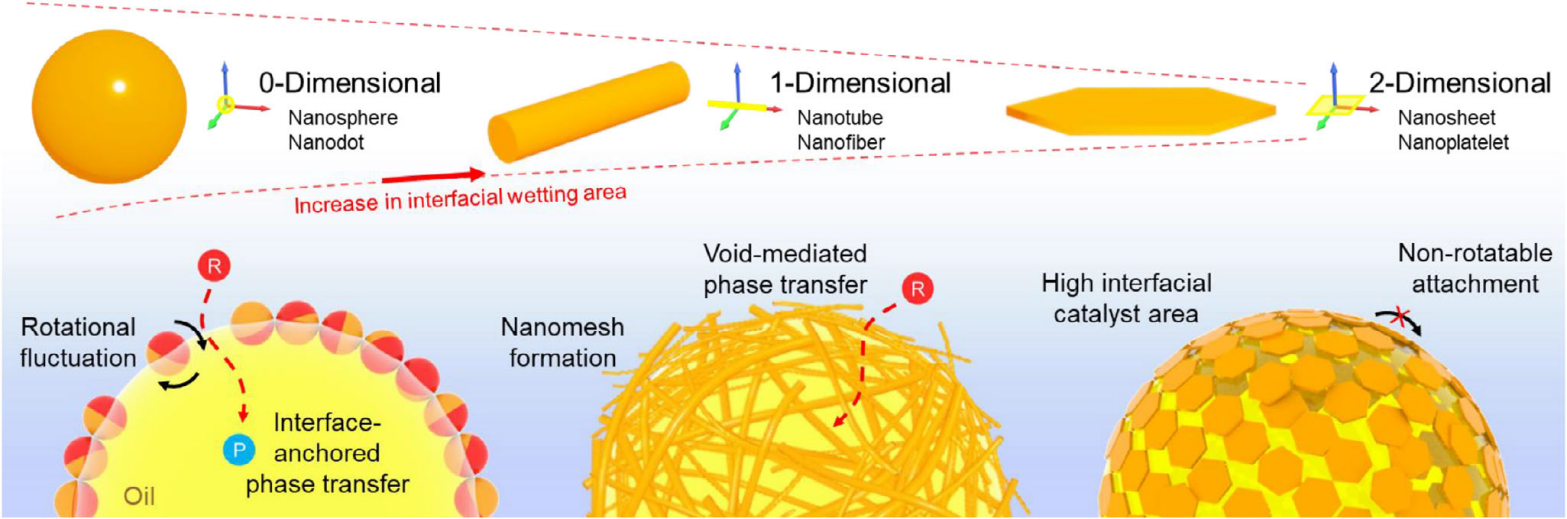
Schematic illustration of the effect of particle shape on interfacial wetting and catalytic function at oil/water interfaces.

In PEMs, solid particles play dual roles as droplet stabilizers and catalyst supports. The resulting microreactor properties are governed not only by surface chemistry but also by colloidal morphology at L–L boundaries. Although all particles are physically three‐dimensional objects, the notations, zero‐dimensional (0D), one‐dimensional (1D) with extended axis, and two‐dimensional (2D) extended lateral dimensions, used here refers to the dominant geometrical anisotropy that controls interfacial adsorption and assembly [9]. Increasing anisotropy systemically alters interfacial packing, contact‐line length, mechanical stress distribution, and transport pathways across the colloidal film (Figure 3a) [79, 80].

### 3.1 Zero‐Dimensional Particles for Wettability‐Driven Stabilization

**FIGURE 4:**
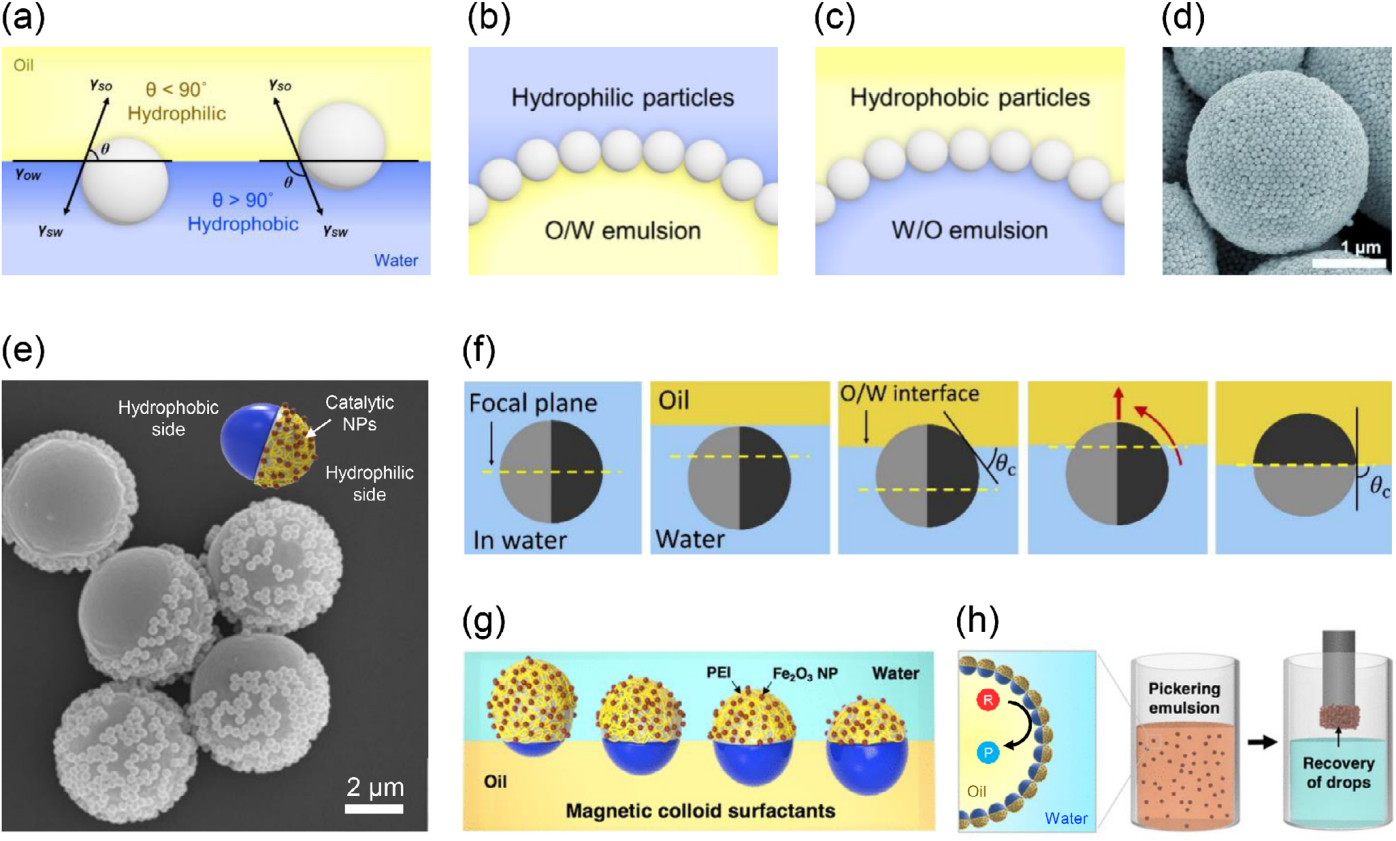
0D colloidal particles assembly for PEM fabrication. (a) Three‐phase contact angle (*θ*) at the oil water interface. (b,c) Preferential emulsion stabilization according to the wettability: (b) hydrophilic particles and (c) hydrophobic particles. (d) Electron microscopic image of spherical particle‐stabilized colloidosomes. Adapted with permission [84]. Copyright 2015, Wiley‐VCH. (e) Electron microscopic image of Janus 0D colloids. Reproduced with permission [85]. Copyright 2016, Wiley‐VCH. (f) Schematic illustration of rotational fluctutation at the oil‐water interface. Adapted with permission [86]. Copyright 202, American Chemical Society. (g) Interfacial orientation based on Janus balance of Janus 0D colloids. (h) Retrievable PEMs designing using Janus particles. Reproduced with permission [87]. Copyright 2018, American Chemical Society.

The key physical parameter governing Pickering emulsion formation is the three‐phase contact angle (*θ*) of the particle at the oil‐water interface (Figure 3b). The adsorption energy (*△E_ads_*) scales with particle radius (*r*), interfacial tension (γ), and *θ* according to:

(3) $$\Delta E_{ads}\approx -\pi r^{2}\gamma {\left(1\pm \left|\cos\theta \right|\right)}^{2}$$

which becomes maximal when *θ ≈* 90°, where the particle wetted equally by both oil and water phases [81]. Hydrophilic particles (*θ* < 90°) preferentially stabilize oil‐in‐water (O/W) emulsions, while hydrophobic particles (*θ* > 90°) favor water‐in‐oil (O/W) configuration (Figure 4a–c). From a catalytic standpoint, the emulsion type determines substrate localization and active site exposure. These wettability rules have been established most clearly for 0D spherical particles, which provide a geometrically simple platform where *θ* and particle size can be varied independently [82]. This makes 0D particles the natural standing point for understanding how wettability translates into micrereactor structure and funcion before advancing more complex 1D and 2D morphologies. Spherical particles assemble into densely packed, often hexagonally ordered monolayers at the droplet surface (Figure 4d) [83].

Wettability‐controlled assembly of isotropic particles provides a conceptual basis for solution‐phase Janus colloid design (Figure 4e). The spherical Janus particles are not completely static after adsorption at L–L interfaces, but can undergo interfacial reorientation as they relax toward the preferred configuration. The rotational adjustment toward equilibrium continuously restores phase‐matched exposure of the hydrophilic and hydrophobic faces, preventing the catalytic patch from remaining buried in a poorly wetted state (Figure 4f) [88]. This can facilitate reactant‐product partitioning from the three‐phase reaction zone. At L–L boundaries, particles whose surface energy lies between that of two heterogenous liquids spontaneously adsorb on the interface and adopt an immersion depth set by *θ* [89]. The immersion geometry defines how much of each hemisphere is exposed to each phase. This sets “Janus balance,” analogous to the relative hydrophilic‐lyphophilic balance (HLB) of molecular surfactants that control emulsion type and stability (Figure 4g) [90, 91, 92, 93]. Janus modification at L–L boundaries enables precise spatial localization of catalytic active site with well‐controlled patch size and wettability contrast. Using this architecture, Fe_2_O_3_ nanoparticles were select ively deposited on the hydrophilic hemisphere of spherical particles, while Pd or Ag nanoparticles were distributed over the particle surface [94]. This produces amphiphilic Janus colloid surfactants that combine catalyltic and magnetic funcions driven by their built‐in wettability contrast [87]. Assembled at the oil‐water interface, they catalyze aerobic oxidation of 4‐methoxybenzylalcohol to 4‐methoxybenzaldehyde with high yield turn over, while the Fe_2_O_3_ patch enables magnetic recovery and recycling (Figure 4h).

### 3.2 One‐Dimensional Particles for Hierarchical Assembly

**FIGURE 5:**
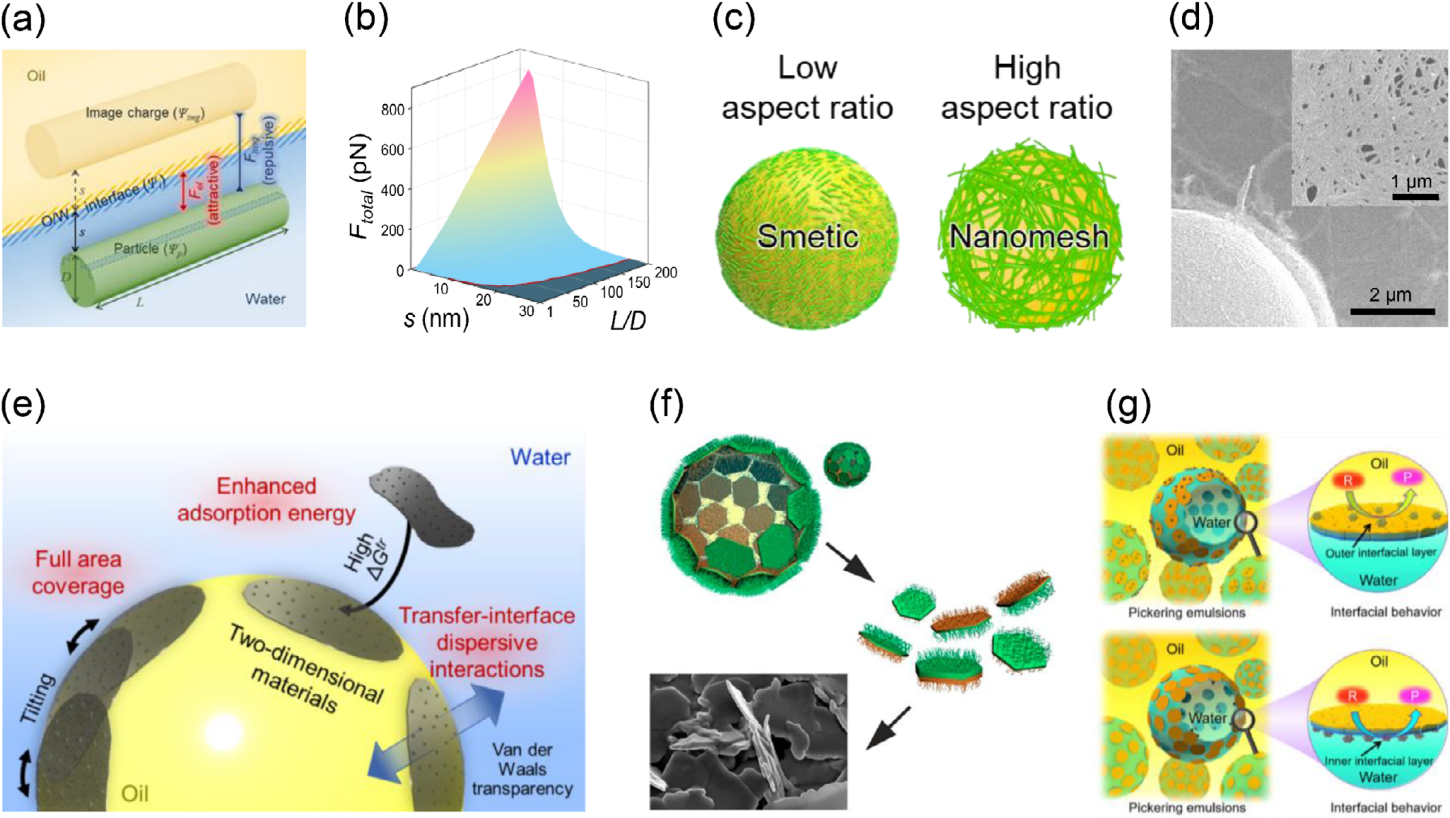
Colloidal dimensionality engineering for PEM fabrication. (a‐d) 1D particle assembly: (a) parallel positioning of a nanorod at L–L boundary and (b) attractive force as function of particle extension. Adapted with permission [98]. Copyright 2025, Wiley‐VCH. (c) Illustration of different aspect ratios of 1D particles‐stabilized Pickering droplets. Reproduced with permission [97]. Copyright 2016, American Chemical Society. (d) Electron microscopic image of nanomesh‐based Pickering particles. Adapted with permission [98]. Copyright 2025, Wiley‐VCH. (e‐g) 2D particle assembly: (e) thermodynamics and molecular barrier properties. Reproduced with permission [105]. Copyright 2014, American Chemical Society. (f) Janus nanoplatelets for PEM stabilization. Adapted with permission [106]. Copyright 2014, American Chemical Society. (g) Spatially‐controlled catalyst incorporation. Adapted with permission [49]. Copyright 2023, American Chemical Society.

The elongated geometry of 1D particles increases the length of the three‐phase contact line along the anisotropic axis, favoring configuration in which the long axis lies parallel to the L–L boundary (Figure 5a) [95]. Above the critical aspect ratio, the side‐on orientation always outcompetes end‐on adsorption, allowing the extended axis to displace a larger area of L–L contact [96]. This preferential positioning is directly reflected in *ΔE_ads_*, which for rod‐like particles scales with length (*l*), width (*d*), and *θ* as:

(4) $$\Delta E_{ads}\approx -ld\gamma {\left(1\pm \left|\cos\theta \right|\right)}^{2}$$

becoming linearly proportional to particle extension at fixed width [97]. Extending the rod axis deepens the adsorption well and enlarges the lateral capture radius over which rods are trapped (Figure 5b) [98]. This generates substantially higher adsorption energies and larger capture distance than for isotropic particles of comparable volume, making 1D colloids more likely to resist desorption under physicochemical stress [99, 100]. For instance, halloysite nanotubes (HNTs) adsorb side‐on at the oil‐water interfaces, where their cylindrical geometry and moderately hydrophilic character give an ≈10^4^ *k_B_T* attachment energy per tube [101]. This value exceeds thermal energy by four orders of magnitude, rendering adsorption effectively irreversible. HNT‐stabilized PEMs therefore exhibit high yield stress and tolerate high internal oil fractions, favorable for repeated operation and easy phase separation [102]. In practice, HNT‐stabilized emulsions have been used as Rh‐catalyzed hydroformylation of long‐chain olefins, converting dodecene to tridecanal [103, 104].

Beyond wettability, anisotropic particle adsorption provides steric hindrance that inhibits Ostwald ripening and coalescence by creating a physical barrier at the L–L boundaries [107]. Rod‐like geometries achieve higher coverage than spherical particles through their ability to reorient and maximize exposed area [108]. Capillary interactions drive end‐to‐end chaining into aggregates that organize into 2D smectic and columnar packed phases. At higher aspect ratios, the assemblies form fibrillar or tubular scaffolds with nanosized voids wrapping around droplets (Figure 5c) [109, 110]. The resulting nanomesh distributes mechanical stress and creates semi‐permeable pathways for selective mass transport across. High‐aspect‐ratio nanocellulose embodies these advantages, generating attractive force two orders of magnitude stronger than spherical particle [98]. The favorable geometric energy configuration overcomes image‐charge repulsion between the pemittivity‐mismatched liquids, driving spontaneous nanofiber assembly into a robust nanomesh with sub‐50‐nm pores (Figure 5d). The nanopores successfully shuttle reactants and products across the semi‐permeable network, leading to accelerated catalysis with high conversion.

### 3.3 Two‐Dimensional Particles for High Atom Economy Engineering

2D colloidal particles position nearly all of their atoms directly at the L–L boundary, allowing the entire solid surface to participate in droplet stabilization. For thin plates with negligible edge area, the minimum free‐energy state orients them parallel to the boundary plane [111]. Unlike spheres, thin plates cannot shift their position to take an upright configuration, as due to the presence of a high‐energy barrier between the two in‐plane position (orientation angle ≈ 0 and 180°) [111]. The occupied area thereby equals the plate surface area, and *ΔE_ads_* for a flat‐lying nanoplate with radius (*R*), oil‐particle interfacial tension (*σ_o_*), and water‐particle interfacial tension (*σ_w_*) is given by:

(5) $$\Delta E_{ads}\approx -\pi R^{2}\left[\gamma -{\left|\left(\sigma_{o}-\sigma_{w}\right)\right|}^{2}\right]$$

which scales with the projected face area [105]. This geometry provides greater stabilization per particle than spheres, thus a small amount of 2D suffices to cover large interfacial areas and passivate droplets against coalescence (Figure 5e) [112, 113]. Graphene oxide (GO) nanosheets exemplify enhanced durability through interfacial multilayer formation. Initial sheets adsorb parallel to emulsion boundaries, forming imperfectly tilted monolayer that leaves nanoscale bare interface. Successive layer continues to occupy residual area, yielding irreversely attached multilayer films [114, 115]. Such co‐continuous shells act as molecular barriers that retard inner‐phase evaporation and solute transport compared with surfactant‐ and isotropic particle‐stabilized systems [116, 117, 118]. The 2D multilayers simultaneously function as mechanical stabilizers through steric passivation of exposed interfacial area.

Amphiphilic 2D particles enable controlled monolayer assembly through wettability contrast across the thickness of nanoplatelets [119]. When one face prefers water and the opposite face prefers oil, the particles adopt a well‐defined orientation and become effectively locked into a lateral, face‐on configurations. In PEMs, this produces thin, elastic interfacial monolayers, in which catalytic sites can be selectively positioned toward either the aqueous or organic side (Figure 5f) [105]. Such price spatial placement within a flat 2D film shortens diffusion paths, maximizes density of active sites per unit area, and more preferentially drives the reaction forward [73]. Pd‐catalyzed Janus silica nanosheets have exploited this strategy to achieve enhanced chemoselectivity in the hydrogenation of *p*‐chloronitrobenzene to *p*‐chloroaniline, maintaining high activity over repeated emulsification‐separation cycles (Figure 5g) [49].

## 4 Surface Functionalization of PICs for Enhanced Catalysis

**FIGURE 6:**
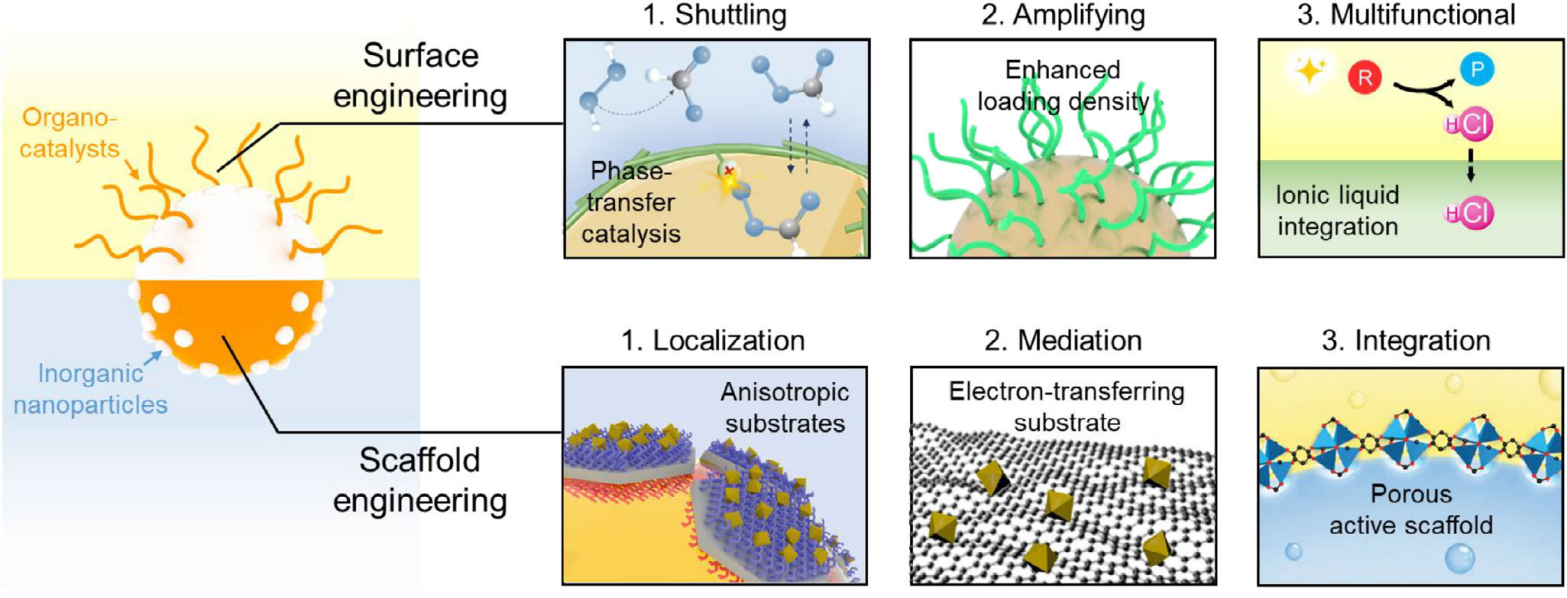
Schematic illustration of organic and inorganic catalysts functionalized PICs and their representative catalytic advantages at liquid–liquid interfaces.

Functionalization is central to PIC design because catalytic performance in PEMs depends not only on catalyst identity, but on how reactivity is organized at the liquid–liquid boundary. In PEMs, functionalization is valuable only when it actively governs interfacial reactivity rather than merely immobilizing a catalyst. In this context, organic functionalization acts primarily through molecular programming, whereas inorganic functionalization operates through scaffold engineering. Together, they define how catalytic sites are accessed, sustained, and translated into interfacial reaction efficiency (Figure 6).

### 4.1 Organic Functionalization of PICs for Catalytic Transport and Reactivity

**FIGURE 7:**
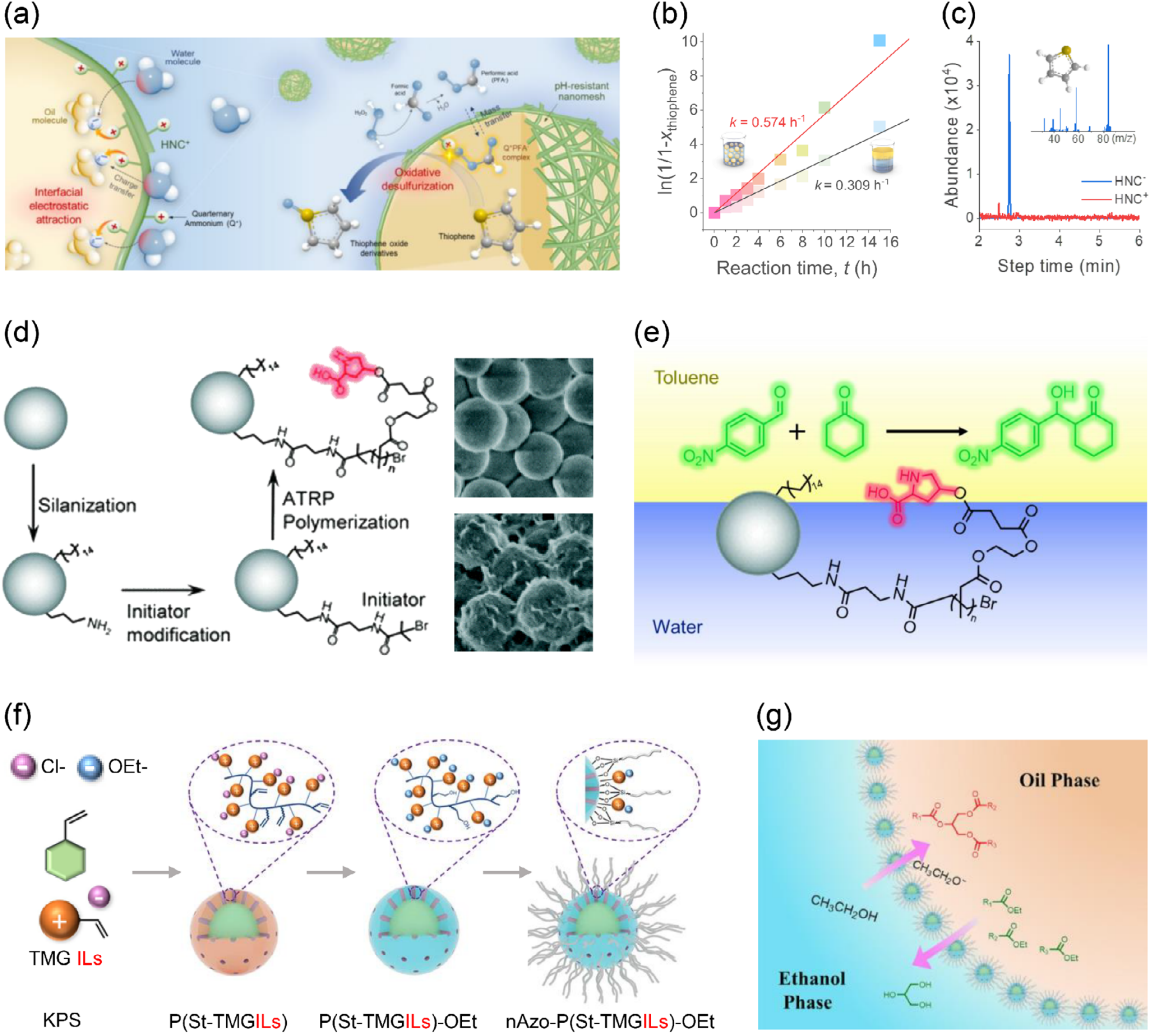
Organically functionalized PICs. (a,b) Molecule‐shuttling strategy: (a) cationic cellulose modified with GTAC as phase‐transfer catalysts at oil‐water interfaces for oxidative desulfurization. (b) Comparative catalytic activity of cationic versus anionic cellulose and (c) reaction kinetics comparing biphasic bulk and PEM systems. Adapted with permission [98]. Copyright 2025, Wiley‐VCH. (d,e) Local density‐enhancing strategy: (d) fabrication of proline‐functionalized polymer‐grafted silica particles and (e) aldol reaction between cyclohexanone and p‐nitrobenzaldehyde at the PE interface. Reproduced with permission [122]. Copyright 2022, Royal Society of Chemistry. (f,g) IL‐integration: (f) light‐responsive ionic liquid‐modified PEs featuring TMG basic catalysts with terminal azo groups for photo‐responsive nanoparticles. (g) Schematic illustration of PEM stabilized by poly(IL)s‐functionalized microspheres for transesterification reactions in biodiesel production. Adapted with permission [123]. Copyright 2023, Elsevier B.V.

Conventional organocatalysts are typically designed to operate in homogeneous media, where molecular dispersion maximizes catalyst‐substrate contact but makes catalyst separation and recovery intrinsically difficult after reaction [120]. This limitation becomes more pronounced in biphasic systems, where catalytic efficiency is governed by whether reactive species can be recruited and concentrated at L–L boundary. The most fundamental role of organic functionalization is to overcome the transport barrier inherent to biphasic catalysis. Quaternary ammonium compound (QAC)‐modified cellulose exemplifies this strategy by converting the stabilizing particle into an ion‐shuttling phase‐transfer catalyst at the oil–water boundary (Figure 7a) [98]. In oxidative desulfurization, cationic cellulose extracts anionic performic acid (PFA), which otherwise cannot migrate spontaneously into the oil phase where oxidation proceeds. The transported PFA then oxidizes thiophene to thiophene oxide, which subsequently returns to the aqueous phase [121]. When cationic and anionic celluloses were compared directly, catalytic activity was observed only for the cationic component, confirming that the interfacial transport function, rather than particle adsorption alone, governs the reaction outcome (Figure 7b). Compared with the corresponding free QAC in bulk biphasic system, the PEM architecture enhance reaction constant from 0.309 to 0.574 h^−1^, showing that organic PICs can couple phase transfer and interfacial area amplification in a single design (Figure 7c).

Once reactant delivery is enabled, the next limitation becomes the local density of catalytic sites at the interface. This is especially relevant for organocatalysts, which often require substantial loading to achieve practical activity [124]. In PEMs, however, the total amount of PIC added does not directly translate into catalytic efficiency, because only the fraction adsorbed at the interface actively participates in reaction [51, 125]. One solution is to raise catalyst density per interfacially active particle [126]. Proline‐functionalized polymer‐grafted silica particles illustrate this concept. After brominating silica nanoparticles to initiate atom transfer radical polymerization (ATRP), proline‐bearing monomers were polymerized from the surface to generate a densely functionalized organocatalytic shell (Figure 7d,e) [127]. In the aldol reaction between cyclohexanone and p‐nitrobenzaldehyde, these proline‐decorated PICs delivered approximately 15‐fold higher conversion than the same amount of free proline, demonstrating that interfacial catalyst crowding can convert molecular organocatalysts into much more efficient heterogeneous PEM catalysts [122]. Moreover, even catalysts that are intrinsically efficient at low loading can benefit when immobilized at the liquid–liquid boundary. This is well illustrated by 2,2,6,6‐tetramethylpiperidine‐1‐oxyl (TEMPO), a widely used catalyst for Anelli–Montanari oxidation that normally operates with comparatively low catalyst quantities [128, 129, 130, 131]. TEMPO‐functionalized snowman‐shaped Janus particles showed higher conversion in the oxidation of cinnamyl alcohol to cinnamaldehyde than bare TEMPO under comparable conditions [132]. Because TEMPO is already catalytically efficient in homogeneous operation, this enhancement is unlikely to arise simply from catalyst loading. Rather, it indicates that positioning TEMPO at the immiscible boundary improves access to reactants from both phases and strengthens phase‐transfer‐assisted catalysis. In other words, the PEM interface acts not merely as a support, but as a reaction field that intensifies catalysis even for intrinsically active organocatalysts.

A further level of sophistication is reached when the organic functionality no longer serves only as a tethered catalyst, but also reshapes the physicochemical behavior of the interface itself. IL‐modified PICs represent this more integrated strategy. As discussed in the Introduction, ILs can promote biphasic catalysis either by directly participating in catalysis or by shifting equilibrium through byproduct removal. When grafted onto Pickering particles, these features can be combined with interfacial stabilization and stimulus responsiveness. In one example, polystyrene microspheres were functionalized with alkaline tetramethylguanidine (TMG)‐based ILs and terminated with azo groups, producing light‐responsive PEM stabilizers for biodiesel transesterification (Figure 7f,g) [123]. IL moieties act as basic catalytic sites, while the modified particle surface simultaneously governs emulsion formation and photoresponsive behavior. This design marks a conceptual transition from catalyst immobilization to interfacial reaction programming, where catalytic function, emulsification, and external control are integrated within a single organic PIC platform.

### 4.2 Inorganic Functionalization of PICs for Scaffolded Interfacial Catalysis

Inorganic catalysts are attractive components for PIC design because they offer high intrinsic activity, thermal robustness, and chemical durability. However, their performance at L–L interfaces depends on how efficiently active sites are exposed, electrically or structurally connected, and stabilized against aggregation or deactivation [133, 134, 135]. Thus, inorganic functionalization in PEMS should address key bottlenecks:1) directional exposure of catalytic sites to the reactive phase, 2) efficient charge or mass transport across the interface, and 3) high‐density integration of stable catalytic motifs within a robust scaffold.

**FIGURE 8:**
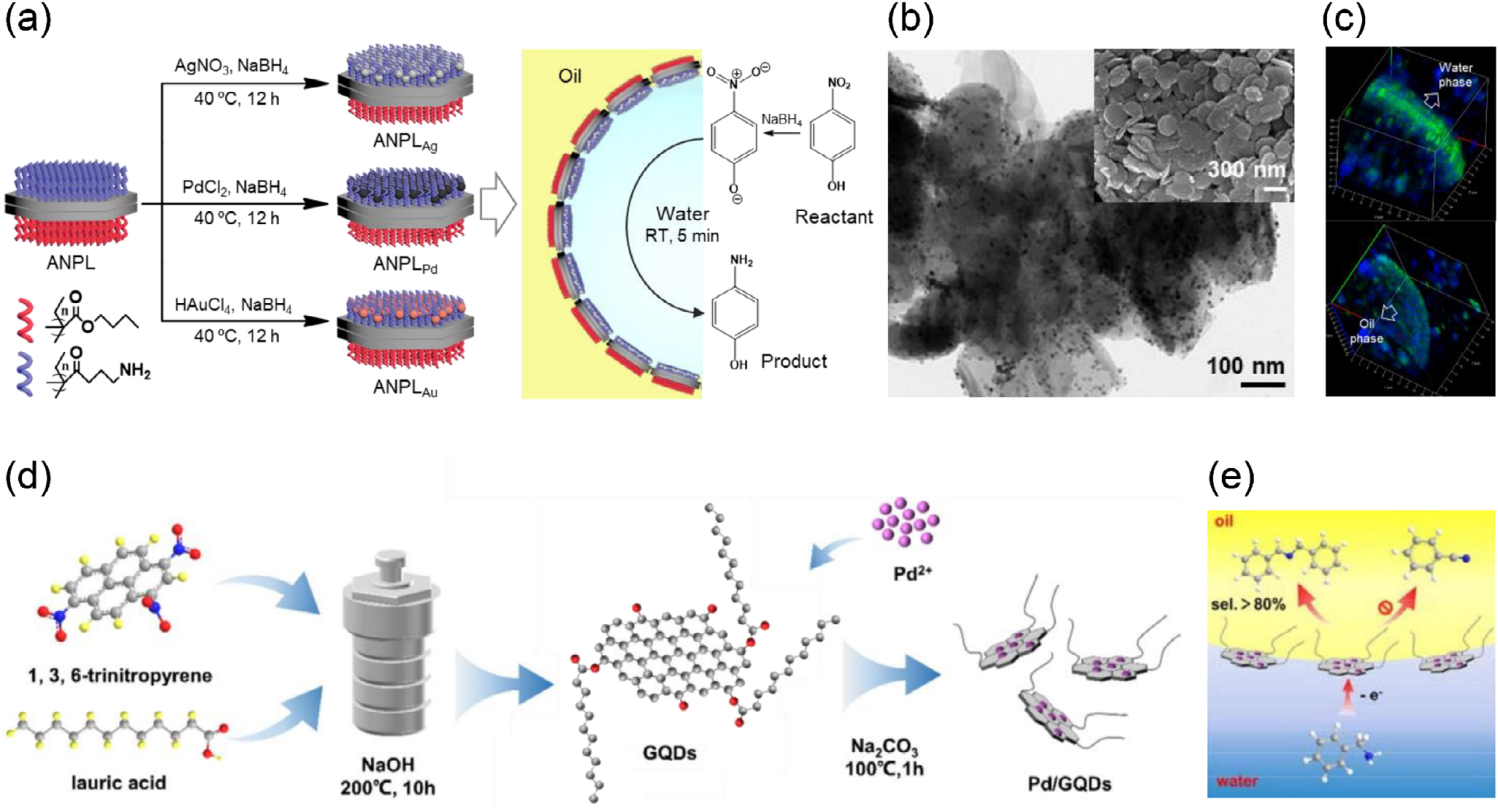
Inoganically functionalized PICs. (a–c) Anisotropic substrates: (a) synthesis of ANPL through selective metal deposition (Ag, Pd, Au) on one face for 4‐NP reduction, (b) electron microscopic images of ANPLs, and (c) confocal laser scanning microscopic images of ANPLs at the oil‐water interface. Adapted with permission [73]. Copyright 2022, Wiley‐VCH. (d, e) Electron‐transferring susbstrates: (d) synthetic pathways of GQDs and (e) schematic illustration of GQD‐stabilized PEMs for benzylamine electroxidation. Reproduced with permission [136]. Copyright 2025, Elsevier B.V.

Directional exposure is most clearly achieved with anisotropic inorganic substrates. Two‐dimensional supports are particularly advantageous because their high surface area‐to‐volume ratio and mechanical robustness allow catalytic species to be deposited selectively on one face while preserving a well‐defined interfacial orientation. Janus zirconium hydrogen phosphate platelets (ANPLs) exemplify this concept (Figure 8a,b) [73]. Once adsorbed at the oil–water boundary, the metal‐decorated face is directed almost entirely toward the reactive phase, as visualized by microscopy of the PEM structure (Figure 8c). In the reduction of 4‐nitrophenol by sodium borohydride, this face‐selective catalyst presentation translated into reaction rates 7.5‐fold higher than those of conventional 4‐NP reduction systems, underscoring how atom efficiency in PEMs depends on whether catalytic sites are exposed rather than buried within the interfacial layer.

A different advantage emerges when the inorganic scaffold is used to mediate transport, especially in reactions coupled to electron transfer. Graphene quantum dots (GQDs) are well suited to this role because they combine high surface area, good conductivity, and resistance to harsh reaction environments [137, 138]. When palladium nanoparticles were grown uniformly on the GQD surface, the resulting composite PIC provided both abundant anchoring sites and an electronically conductive framework (Figure 8d) [136]. In this architecture, the GQD support suppresses Pd aggregation while facilitating charge transfer during catalysis. As a result, benzylamine electrooxidation proceeded efficiently in the Pickering microreactor, illustrating that conductive inorganic supports can do more than immobilize catalysts: they actively couple interfacial catalysis with transport across the reaction field (Figure 8e).

This logic reaches its most integrated form in porous crystalline scaffolds, where the support itself becomes part of the catalytic microenvironment.Metal‐organic frameworks (MOFs) are crystalline porous solids constructed from metal nodes and organic linkers, offering exceptionally high surface areas and precisely tunable chemical environments [139, 140, 141]. In PEMs, this modularity allows MOF nanoparticles to function not merely as catalyst carriers, but as interfacially active catalytic solids capable of supporting reactions from simple condensations to multicomponent tandem transformations [142, 143]. A representative example is zeolitic imidazole framework‐90 (ZIF‐90) moduified with 1,4‐diaminobutane and N,N‐dimethylacetamide dimethylacetal, which introduces strongly basic amidine groups that serve both as emulsifier and catalytic sites. n these ZIF‐90‐stabilized PEMs, Knoevenagel condensation proceeds efficiently because the amidine sites deprotonate malononitrile and promote nucleophilic addition to aldehydes at the interface, affording α,β‐unsaturated dicyano products under mild conditions over 98% conversion efficiency within a hour [144]. Taken together, the organic and inorganic functionalization strategies demostrate that the superior performance of PEMs over bulk catalysis is governed not only by catalyst identity, but by how precisely active sites are organized, accessed and sustained at the L–L interfaces (Table 2).

**TABLE 2:** Comparative catalytic performance of PEMs and their conventional bulk counterparts.

Organic reaction	PEM system discussed here		Bulk counterpart		Relative improvement	Refs.
	Stabilizers	Kinetics index	Catalysts	Kinetics index		
Oxidative desulfurization	Cationic nanocellulose	Rate constant, *k* = 0.574 h^−1^	QAC in biphasic bulk	Rate constant, *k* = 0.309 h^−1^	1.86	[98]
Aldol reaction	Proline‐functionalized nanoparticles	Conversion efficiency, 93.2%	Free proline	Conversion efficiency, 6%	15.5	[122]
Aneli oxidation	TEMPO‐anchored nanohybrid particles	Reaction time, 62.5 min (>90%)	Cu‐nitroxyl‐TEMPO	Reaction time, 600 min (>90%)	9.6	[145]
Oil transesterification	Poly(IL)s‐functionalized microspheres	Rate constant, *k* = 0.00429 min^−1^	Zr/CaO	*Rate constant, *k* = 0.00378 min^−1^	1.13	[146]
4‐NP reduction	Metal nanoparticles‐anchored nanoplatelets	Turnover frequency, 0.032 mol·h^−^1·g^−1^	Free CuO nanoparticles	Turnover frequency, 0.0084 mol·h^−^1·g^−1^	3.81	[147]
Benzylamine electrooxidation	Pd/GQDs	Conversion efficiency, 45%; selectivity, 81%	Free Pd nanoparticles	Conversion efficiency, 6.6%; selectivity, 21%	Conversion efficiency, 6.81; selectivity, 3.85	[136]
Knoevenagel condensation	Amine‐modified ZIFs	Reaction time, 1 h (>98%)	Free ZIFs	Reaction time, 3 h (>98%)	3	[144]

* Calculate from Arrhenius parameters: activation energy (*E*_a_) = 42.5 kJ mol^−1^, (*A*) = 2.2 × 104 min^−1^, and the rate constant (*k*) at 75°C = 0.0123 min^−1^. Reproduced with permission [146].

### 4.3 Biological Functionalization of Pickering Stabilizers as PIC

**FIGURE 9:**
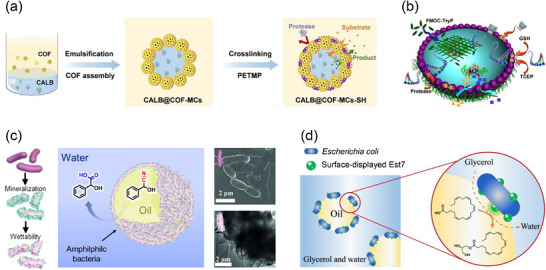
Biologically‐derived and ‐modified PICs. (a) CALB immobilized COF. Adapted with permission [157]. Copyright 2023, Wiley VCH. (b) Enzyme‐immobilized proteinosome. Adapted with permission [161]. Copyright 2014, American Chemical Society. (c) Wettability‐controlled bacteria. Reproduced with permission [162]. Copyright 2015, Wiley VCH. (d) Surface‐displayed esterase (Est7) on Escherichia coli for biodiesel reactions. Adapted with permission [163]. Copyright 2024, American Chemical Society.

Enzymes lose their catalytic activity in organic solvents primarily because the solvents disrupt the crucial hydration shell of the protein, leading to conformational denaturation [148, 149]. This structural change deforms the enzyme's active site, preventing effective substrate binding and reaction [150]. Therefore, recent studies are using porous structures, covalent organic framework (COF) to immobilize the enzymes to sustain their activation [151, 152, 153]. When enzyme‐immobilized COFs are positioned at the interface, they can directly contact hydrophobic moieties while being protected from deactivation caused by organic solvent exposure, achieving high substrate concentration in the vicinity of the active sites [154, 155]. Particularly, lipases and esterases undergo “interfacial activation” at the interface which induces conformational changes such as opening of the lid domain, exposing the catalytic site and increasing turnover rates by 19‐fold higher than free lipase [156]. This study synthesized Candida Antarctica lipase B (CALB) containing PIC by cross‐linking covalent organic frameworks and employed them as emulsifiers for esterification of 1‐hexanol and hexanoic acid (Figure 9a) [157, 158, 159]. Similarly, microgels have been widely explored as versatile platforms for enzyme immobilization in Pickering emulsions [160]. A key advantage of microgels is that they can confine enzymes without the need for covalent bonding, which helps preserve enzymatic activity for prolonged periods. In addition, their slight permeability may allow gradual retention and release behavior that contributes to sustained catalytic function and repeated reusability. Because this strategy is not limited to a specific enzyme type, microgels can be regarded as a broadly applicable template and a promising material platform for the construction of multicatalyst systems, as discussed in later sections.

Protein based biocatalysts, proteinosomes, shows mechanically robust and chemically stable compartments that outperform liposomes under harsh conditions [164]. Their tunable semipermeable membranes allow selective transport of small substrates while retaining enzymes or biomolecules, enabling efficient and controlled biocatalysis [165, 166]. Also, interior compartmentalization can be achieved by the gelation of inner phase, which are used for mimicking artificial cells and programmable biochemical systems [167]. They can be fabricated by using amphiphilic protein‐polymer nanoconjugates prepared by grafting poly(N‐isopropylacrylamide) (PNIPAAm) onto bovine serum albumin (BSA). The enzyme‐mediated amino acid dephosphorylations form the proteinsome gel, not only regulating the release of the drugs from the proteninosomes but also self‐healing the outer membrane under heat condition (Figure 9b) [161].

Whole‐cell catalysis employs entire bacterial cells as catalysts, wherein all intracellular enzymes function as active sites [168]. Leveraging this concept, one study applied *E. coli* cells to degrade polycarbonate plastic into bisphenol A within PEMs [169, 170]. Green catalytic degradation of polycarbonate in PEMs offers an atom‐economical alternative to landfilling or incineration, converting plastic waste into high‐value chemicals while reducing carbon footprint and promoting resource sustainability. Another example involved the amphiphilic modification of *Alcaligenes faecalis ATCC 8750* for use as a PIC in the bioconversion of oil‐phase mandelonitrile to hydrophilic mandelic acid. They mineralized the single bacterium instead of immobilizing microbes in supports to improve microbial performance, prevent diffusional limitation blocked by the support (Figure 9c) [162]. Beyond single bacterium, baceteria‐enzyme complexes based PIC was conducted using cell surface‐displayed catalytic approaches. The *Est*7 enzyme exposed on the outer membrane of *E. coli* cells through genetic engineering. These biocomplexes positioned at the interface for esterification reactions between Eicosapentaenoic acid monoglyceride and glycerol (Figure 8d) [163].

## 5 Catalysts Recovery and Recycle Engineering

### 5.1 Stimuli‐Responsive Demulsification for Controlled Phase Separation

**FIGURE 10:**
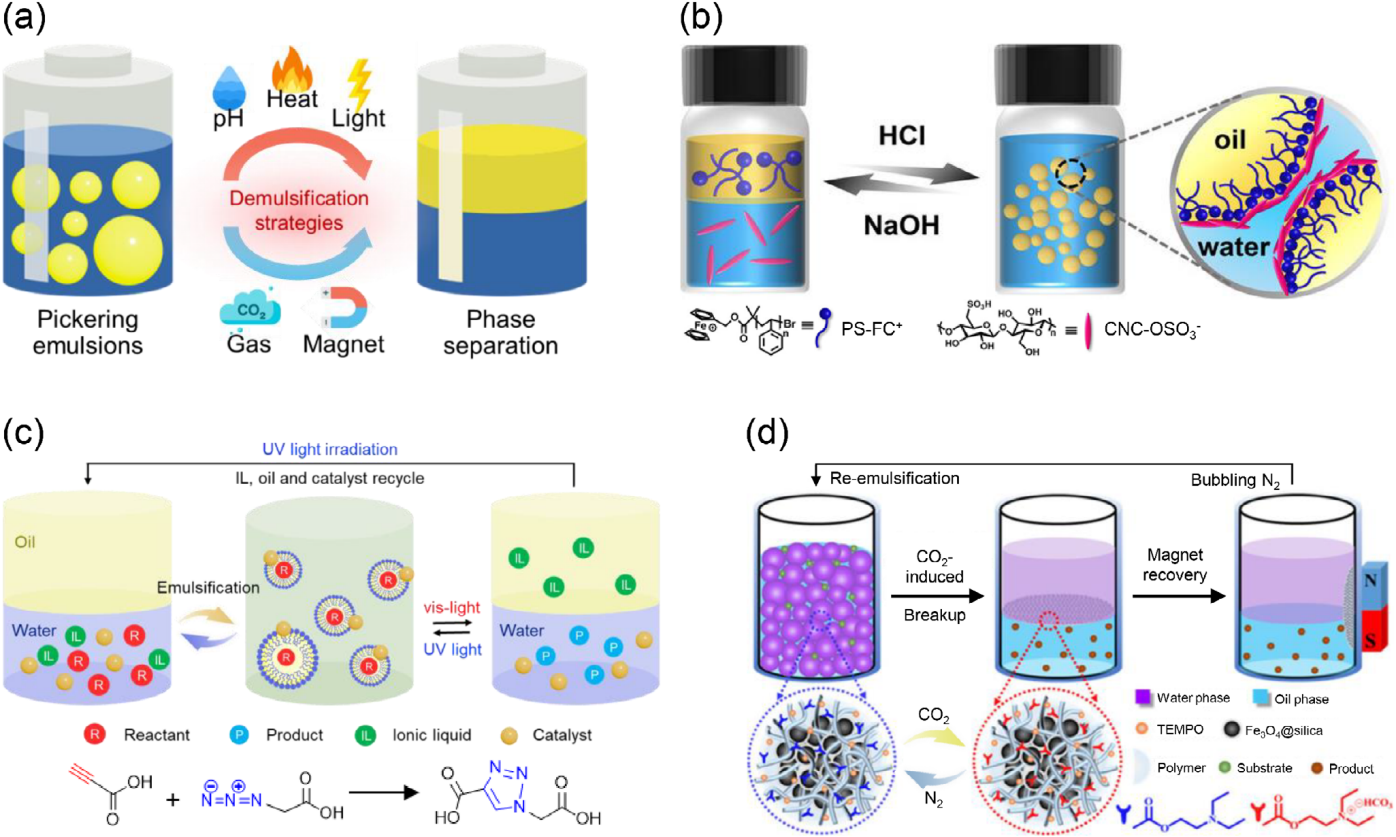
Stimuli‐driven demulsification strategies for catalyst recovery. (a) Overview of external stimuli for in‐situ phase separation. Responsive mechanisms: (b) pH‐switchable coacervate systems. Reproduced with permission [174]. Copyright 2023, Wiley VCH. (c) Photochemical spiropyron isomerization. Reproduced with permission [175]. Copyright 2025, Wiley VCH. (d) CO_2_‐responsive protonation with magnetic retrievability. Adapted with permission [176]. Copyright 2019, American Chemical Society.

In multiphase catalysis, the way in which catalytsts are recovered and reused is a primary determinant of overall process efficiency [59]. Every cycle where catalyst is lost to the product stream, waste phase, or auxiliary separation directly increases non‐productive material consumption and requires additional energy input. The exceptionally high adsorption energy of solid colloids at L–L boundaries means that once assembled, these interfacial layers resist to disruption [171, 172, 173]. Whether a PEM system ultimately reduces waste generation therefore depends on how intgelligently its recovery step is engineered. Stimuli‐responsive systems address this challenge by merging the concepts for catalytic reaction and separation. External triggers temporarily alter particle wettability or inter‐particles interactions, collapsing the emulsion or extracting particles from the PEMs without additional mechanical treatment (Figure 10a).

pH‐responsive PEMs exploit the protonation‐deprotonation of ionizable surface groups to modulate electrostatic interactions and wettability at the oil‐water boundaries [177, 178]. At the pH exhibiting the strongest attractive ionic strength, enhanced binding form dense interfacial layers and stable emulsions. Shifting the pH to weaken binding affinity lowers surface coverage and induces demulsification. A representative design employs sulfated cellulose nanocrystals (CNC‐OSO_3_^−^) that co‐assemble with cationic species into nanoparticle surfactants [174]. CNC‐OSO_3_^−^ electrostatically coacervates with ferrocenium‐terminated polystyrene (Ps‐Fc^+^) at the toluene‐water interface. At pH ≈ 3, strong coacervate ion pairing lowers interfacial tension, producing dense layers that stabillize long‐lived emulsions. Rasing pH toward 9 partially neutralize the sulfate and perturbs the ion pairing, ultimately triggering demulsification as surface coverage falls below the percolation threshold (Figure 10b) [179]. Recent work with Pd‐loaded carboxylated CNCs demonstrates precisely this strategy for catalytic hydrogenation, enabling efficient recovery of both and catalyst across multiple cycles [180].

Thermally switchable platforms rely on polymers with a lower critical solution temperature (LCST) tethered to particle surfaces [181]. Below LCST, the polymer remains hydrated and hydrophilic, it is changed to hydrophobic above LCST. The objective is to conduct catalysis at temperatures where emulsions are highly stable, then cross the LCST to demulsify and separate phases rapidly without chemical additives [182, 183, 184, 185]. For this, thermo‐resposive Janus silica nanosheets (JSNs) are designed in which one face is grafted by thermo‐responsive polymer brushes while the opposite face carries a chiral Ti(salen) complex for asymmetric sulfoxidation [186]. At room temperatue (T < cloud point ≈ 39°C), hydrogen bonding between polymers and water renders the polymeric face hydrophilic, while the Ti(salen) face remains hydrophobic. The resulting anisotropy locks the nanosheets at the oil‐water interface and yields extremely stable emulsions that enhance sulfoxidation rates and conversions. Upon heating above the cloud point, polymer chains collapse and form intra‐brush hydrogen bonding, swithcing the face to a more hydrophobic state. This loss of amphiphilicity causes rapid demulsifiation, and the nanosheets migrate to the organic phase for separation and reuse.

Photo‐switchable Pickering emulsions translate photochemical isomerization of surface ligands into changes in interfacial activity [187, 188, 189, 190]. Spiropyran (SP) becomes hydrophobic and neutral under UV irradiation, while visible lights make SP in reverse form [175]. A spiropyran‐based ionic liquid (SPIL) employed within UiO‐66‐NH_2_‐stabilized PEMs system illustrates this mechanism. Under UV lights, SPIL converts to its merocyanine form, increasing its charge density and affinity for both the UiO‐66 catalyst surface and the oil‐water boundary. This lowers interfacial tension and stabilizes fine emulsions suitable for aqueous CuAAC click reactions yielding hydrophilic products. Subsequent visible light exposure drives back‐isomerization, reducing interfacial acitivity (Figure 10c).

Gas‐switchable systems use Brønsted bases (e.g., tertiary amines) or amidines‐achored particles. Exposure to CO_2_ gas in the prescence of water generates bicarbonate and protonates basic groups, converting hydrophobic surfaces into hydrophilic zwitterionic or cationic ones. This approach employ an inexpensive, abundant and benign trigger that can be fully removed by purging with an inert gas, leaving no persistent additives [191, 192]. This concept is exmplified by Fe_3_O_4_‐incorporated silica nanospheres bearing tertiary amino groups (diethylamine, DEA) on a polymer shell and TEMPO as an immobilized oxidant catalyst [176]. In their native state, the particles are moderately hydrophobic (*θ* ≈ 90°–97°) and stabilize Pickering emulsions for Anelli oxidations. Post‐reaction, bubbling CO_2_ protonates surface of DEA, dramatically increasing hydrophilicity and destabilizing the emulsions. Subsequent N_2_ purging for reverse protonation, it restored the original wettability and enabled reuse over at least five cycles with negligible activity loss (Figure 10d). This system also incorporates superparamagnetic domains for magnetic retrieval. An external magnetic field extracts the catalyst without filtration or centrifugation. Magnetically recyclable Pickering catalysts have been further engineered or density‐matched emulsification, where controlled magnetic content modulates emulsion stability and separation kinetics [193, 194]. Catalysts with optimized density achieved high conversions with rapid phase separation and retain activity across multiple cycles.

### 5.2 Biodegradable Colloids for Sustainable Catalyst Scaffolds

**FIGURE 11:**
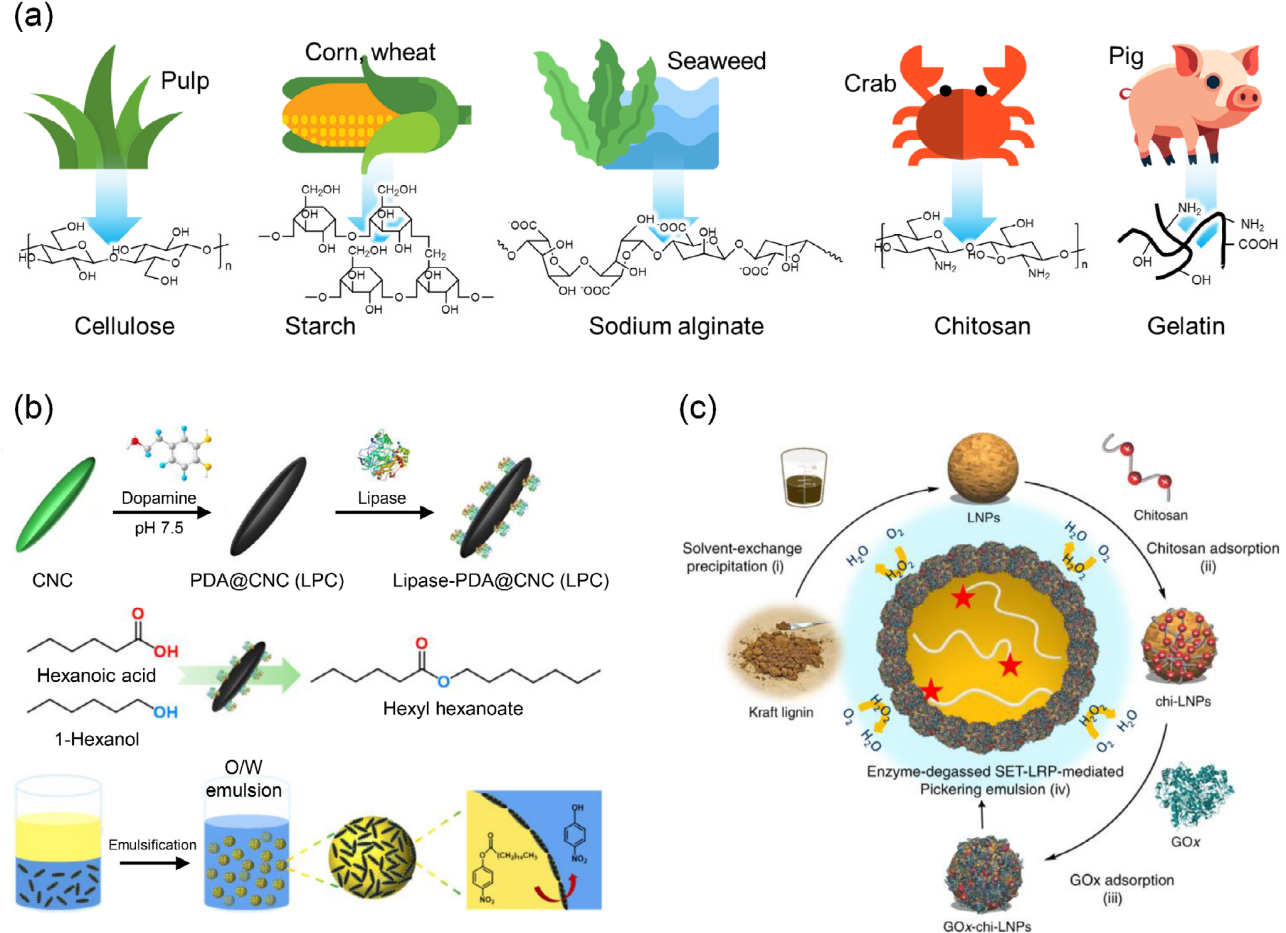
Bio‐friendly degradable stabilizers for enhanced sustainability. (a) Classification of bio‐derived polymers. Polysaccharide‐based colloidal engineering: (b) enzyme‐immobilized CNCs for biocatalysts. Reproduced with permission [202]. Copyright 2024, American Chemical Society. (c) LNPs as aromatic radical scavenging interfacial catalysts. Adapted with permission [203]. Copyright 2020, Springer Nature.

The transition from inorganic or petroleum‐derived solid stabilizers to biodegradable and bio‐derived colloids is central to making PEMs compatible with long‐term environmental expectations. In conventional configurations, the colloidal scaffold is often persistent mineral waste or microplastic particulates [195, 196, 197]. Bio‐derived colloids built from renewable carbon and degradable backbones directly address this end‐of‐life gap (Figure 11a) [198]. They reduce cumulative toxicity and enable carbon recycling into short biogenic cycles. Recent examples show that these soft supports do not trade off catalytic performance. Instead, their high surface area, rich surface chemistry, and intrinsic bio‐functionality often provide unique advantages [199]. Polysaccharide‐based colloids are among the most promising candidates. CNCs and nanofibrils combine abundant, low‐cost feedstock with rod‐like geometry and dense surface hydroxyl groups [200, 201]. They offer excellent water dispersibility and tunablilty, making them ideal carriers for interfacial immobilization of enzymes or organocatalysts. Lipase‐immobilized CNC represents a system where a fully bio‐based colloidal scaffold achieves both high catalytic throughput and facile recovery [202]. Rod‐like CNCs were coated with a thin polydopamine (PDA) layer and immobilized lipase enzymes. The resulting particles exhibit partial wettability toward both aqueous and oil phases and reduced absolute surface potential compared with pristine CNCs. This translates into smaller, more stable emulsions and nearly complete particle adsorption at the L–L boundary. For recovery, the particles form relatively weakly charged large aggregates separable by simple centrifugation. After multiple cycles, immobilized enzymes retain high activity, demonstrating nanocellulose scaffold enables relevant reuse (Figure 11b).

Lignin nanoparticles (LNPs) represent a complementary class that valorize kraft lignin, an underutilized aromatic side stream of pulping, into colloidal stabilizers with inherent redox activity and UV‐shielding properties [204, 205]. Their aromatic structure affords strong interfacial adsorption and additional functionaliity such as radical scavenging, which can be directly exploited in catalytic processes and final products properties [206, 207]. LNPs extend bio‐derived PEMs into aromatic polymerization chemistry. LNPs coated with chitosan and glucose oxidase (GOx‐chi‐LNPs) simultaneously act as Pickering emulsifiers and enzymatic oxygen scavengers in single electron transfer living radical polymerization (SET‐LRP) of hydrophobic vinyl monomers [203]. After polymerization, the LNPs remain uniformly embedded in polymer films, allowing direct melt‐processing of lignin polymer composites without extra compatibilization steps (Figure 11c). This design converts an otherwise waste stream into a multifunctional, bio‐based stabilizer and performance additives, improving both carbon utilization and process sustainability.

## 6 Advanced Microreactor Designs

### 6.1 Finely Architectured Membrane for Enhanced Interfacial Transport

**FIGURE 12:**
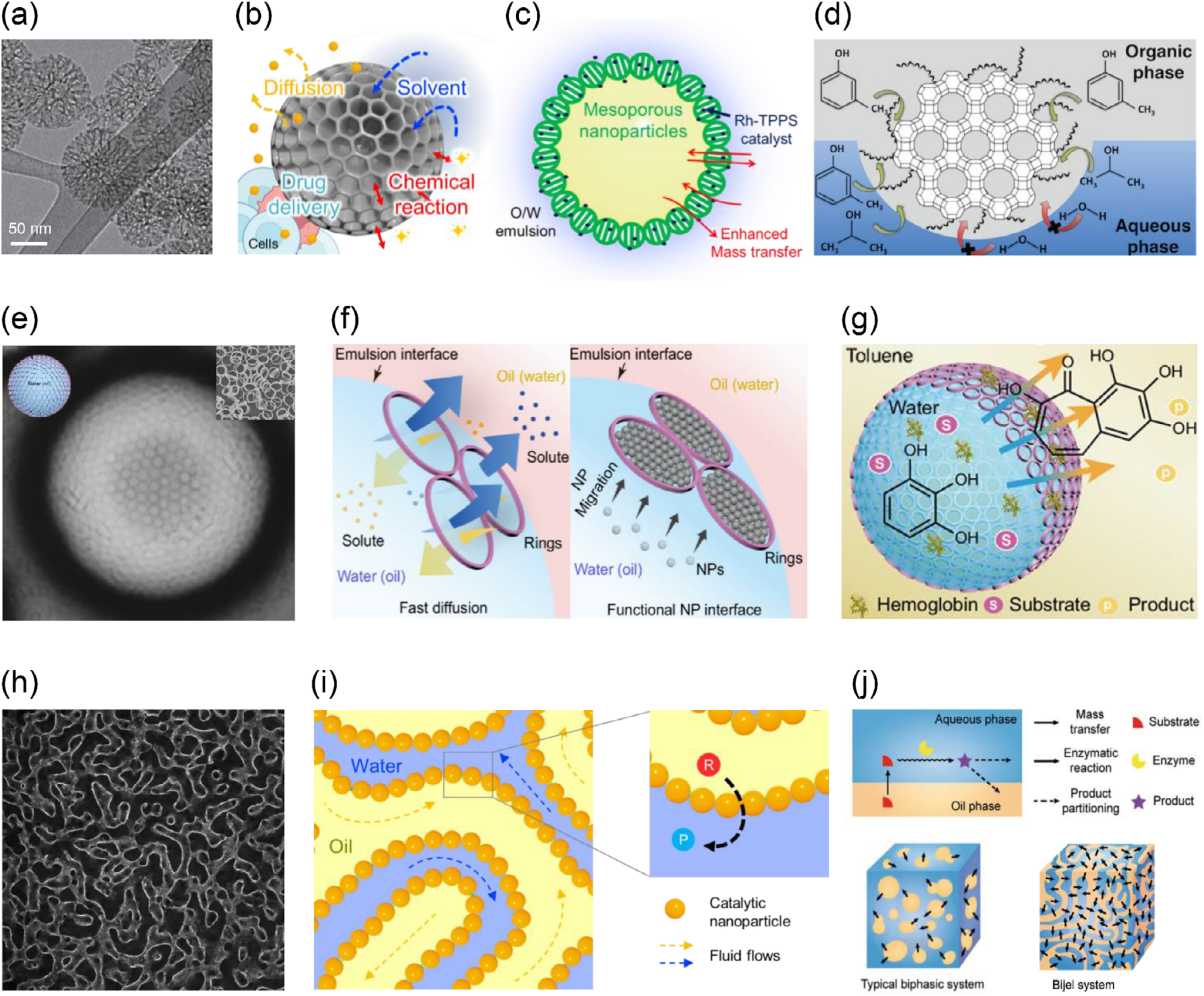
Multi‐dimensional architectures for advanced PEMs. (a‐d) Particle porosity engineering: (a) electron microcsopic images of MSNs. Adapted with permission [215]. Copyright 2016, Elsevier B.V. (b) Enhanced surface interactions and (c) mass transfer pathways of MSNs‐stabilized PEMs. Reproduced with permission [211]. Copyright 2016, Elsevier B.V. (d) Zeolite‐stabilized PEMs for biofuel upgrading reactions. Reproduced with permission [214]. Copyright 2012, American Chemical Society. (e–g) Particle marginal engineering: (e) electron microscopic images of toroidal silica particles at emulsion interfaces, (f) schematic illustration of bidirectional diffusion at the L–L boundary, and (g) PEMs for pyrogallol oxidation and surface‐enhanced Raman spectrocopic sensoring. Adapted with permission [51]. Copyright 2025, Springer Nature. (h–j) Boundary structural engineering: (h) electron microscopic images of bigels, (i) 3D microchannel network in bicontinuous interfacially jammed emulsion gels (bijels), and (j) bijel microreactors for lipase‐catalyzed triglyceride. Adapted with permission [216]. Copyright 2019, Springer Nature.

3D structured particles transcend simple geometries by introducing additional structural features, including internal porosity, complex topology, and enhanced surface area. In these systems, the stabilizer becomes a sophisticated colloidal architecture that shapes how liquids, solutes, and catalysts access the L–L boundary [208, 209]. Mesoporous colloids introduce an internal length scale, with the porous framework controlling transport across the boundary (Figure 12a,b) [210]. They present a high density of pore entrances at the oil‐water contacts, so each adsorbed particle behaves as a cluster of nanochannels connecting the two immiscible liquids. In amphiphilic mesoporous silica nanospheres (MSNs)‐stabilized emulsions, the pore network permits rapid reactant and product diffusion alongside uniform Rh‐TPPTS catalyst distribution (Figure 12c) [211]. Furthermore, inorganic crystalline microporous 3D particles can sustain their structures in harsh conditions with the advantage of porosities [212]. Zeolite affords molecular sieving by screening reactants and products according to their size as they diffuse through the crystalline channels [213]. Octadecyltrichlorosilane‐functionlized zeolites with enhanced thermal stability enable PEM‐mediated phase‐selective acid catalysis for biomass oil refining under elevated temperature without framework collapse (Figure 12d) [214].

Ring‐shaped colloids exploit topology to engineer the interfacial architecture. Toroidal silica particles assemble as dense annular belts around each droplet (Figure 12e). The resulting ring‐based PEMs maintain mechanical stability while exposing most of the bare L–L boundary (>80%). The colloidal torus pins robust contact lines and prevent drop coalescence, yet its low areal coverage minimizes steric blocking and permits rapid bidirectional diffusion (Figure 12f) [51]. This opened interface can then be decorated with catalytic or plasmonic nanoparticles without competition for space with emulsion stabilizers [217]. Ring‐stabilized emulsions demonstrate higher catalytic efficiency than conventional nanosphere‐covered droplets and provide sensitive surface‐enhanced Raman scattering (SERS) microreactors [51]. This design enables dual localization of catalysis and spectroscopic readout within the accessible regions (Figure 12g) [218].

Bicontinuous interfacial jammed emulsion gel (bijel) systems represent the multi‐dimensional limit where the entire L–L boundary converts into a continous, tortuous catalytic membrane (Figure 12h) [219, 220]. Bijel is a structually stable bicontinuous emulsion fabricated by the interfacial jamming of nanoparticles between two immiscible fluids with spinodal decomposition [221]. In contrast to conventional emulsions, neither phase is confined to isolated droplets; rather, both phases form continuous, interpenetrating domains. The intrinsic bicontinuous phase separation generates an exceptionally large interfacial area, while the interconnected architecture enables unimpeded molecular diffusion throughout each phase domain [222]. Consequently, the intertwined oil and water channels establish a three‐dimensional microchannel network with exceptionally high accessible interfacial area. (Figure 12i). In lipase‐catalyzed triglyceride hydrolysis, bijels provide substantially faster mass transfer of the oil‐soluble substrate to the water‐soluble enzyme than a stirred biphasic mixture, yielding significantly enhanced reaction kinetics (Figure 12j) [216]. Nevertheless, bijel fabrication remains technically demanding, and existing studies have been largely confined to template‐mediated approaches wherein polymerization yields solidified architectures for catalytic purposes, rather than leveraging the native liquid–liquid interface [223]. Substantial further investigation is warranted to realize the industrial and academic potential of Bijel systems.

### 6.2 Multicatalyst Microreactor Stabilized by Janus Particles

**FIGURE 13:**
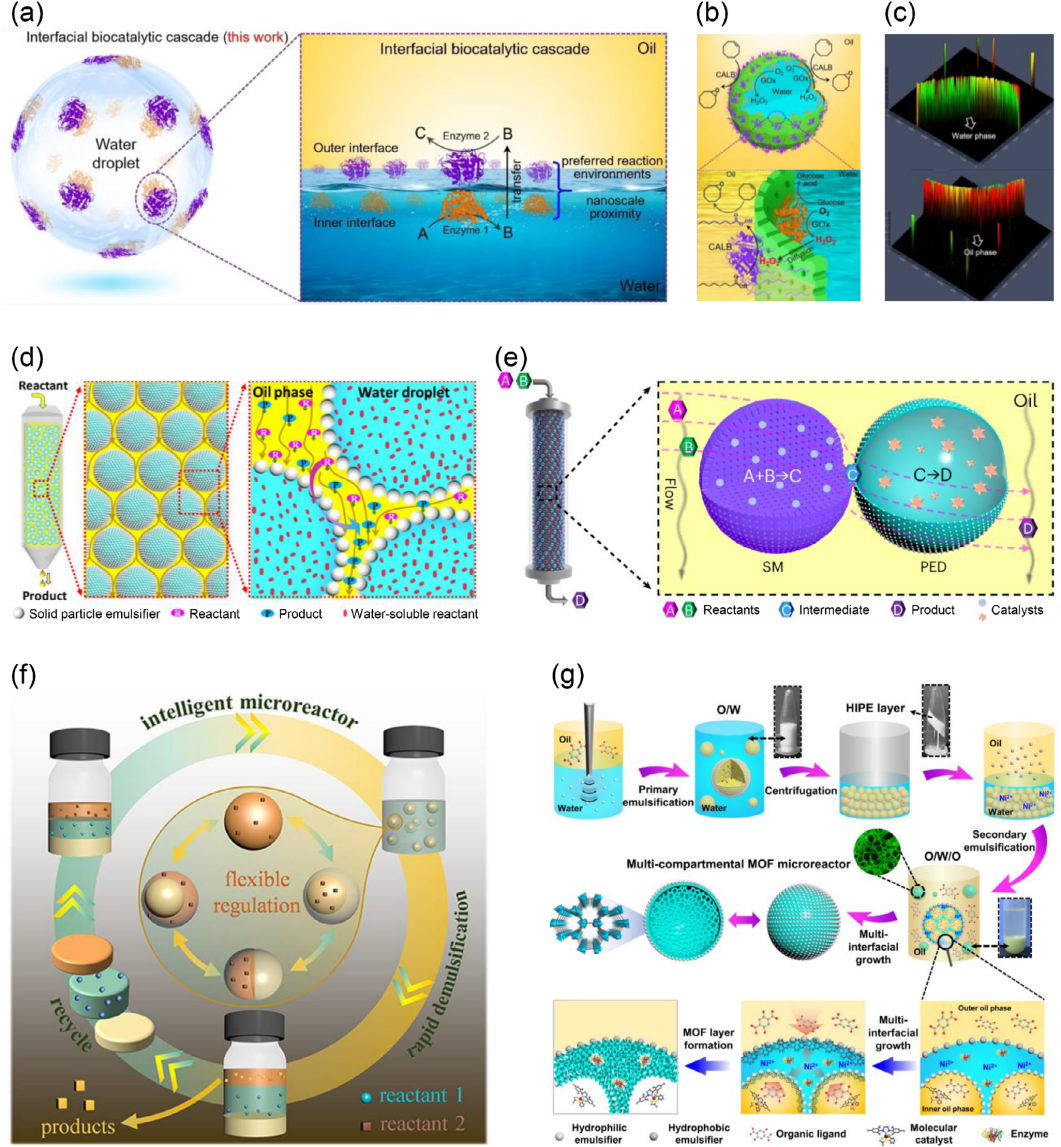
Advanced microreactor designs. (a–c) bifunctional PICs: (a) schematic illustration of Janus particles at the oil‐water interface for multistep catalysis microreactors, (b) reaction pathways, and (c) confocal laser scanning microscopic images of bicatalyst‐immobilized Janus nanoplatelet particles and the schematic illustration of reactions. Adapted with permission [232]. Copyright 2023, Wiley VCH. (d,e) Continuous flow microreactors: (d) Conventional PEMs‐based flow reactor. Adapted with permission [233]. Copyright 2016, American Chemical Society. (e) Solid microspheres‐PEM‐integrated CFMs for cascade reaction. Adapted with permission [234]. Copyright 2024, Springer Nature. (f,g) Double emulsion templated microreactors: (f) mass transfer of reactant between fluorocarbon and hydrocarbon oil. Adapted with permission [235]. Copyright 2024, Wiley VCH. (g) Multi‐compartmental MOF microreactors. Adapted with permission [236] Copyright 2023, Springer Nature.

Executing consecutive or cascade reactions in PEMs fundamentally requires either multiphase systems or multicatalyst configurations. Janus particles which possess chemically or physically compartmentalized sections within single particle represent the most prevalent strategy for achieving multicatalyst systems [224]. The most basic spherical Janus PICs can be synthesized readily via emulsion seeding polymerization followed by catalyst incorporation [225, 226, 227]. Platelet‐shaped Janus particles offer particularly advantageous geometries, providing optimal bilateral separation of catalysts that are conjugated in distinct planes [228, 229, 230]. For instance, PEMs stabilized by 2D Janus particles with CALB and glucose oxidase (GOx) functionalized on opposite sides demonstrated interactive cascade reactions (Figure 13a) [231]. Glucose was oxidized to gluconic acid in the aqueous phase, generating hydrogen peroxide that transferred through the pores to participate in CALB‐catalyzed esterification reactions in the oil phase (Figure 13b,c).

### 6.3 Multiphase Cascade Reaction with Continuous Flow Microreactors

Multiphase microreactors for cascade reaction must be meticulously designed considering the following conditions: 1) high reaction selectivity and solvent affinity of intermediates for next reaction [232, 237], 2) strategic spatial arrangement of catalysts for achieving orthogonality and preventing cross‐interference [238, 239], 3) non‐disruptive byproduct formation from each reaction step [240, 241]. Especially, the spatial arrangement of catalysts in a single phase is the most difficult part for designing. Continuous Flow Microreactors (CFMs) which is packed with diverse types of PEMs in the flow reactors can prevent the inteference of catalysts inevitably coexist in the same phase, whether aqueous or oil. For instance, due to the extremely rapid kinetics of acid‐base neutralization, acid and base catalysts cannot be co‐located in the same phase [242, 243]. By preparing two distinct Pickering emulsions, with acid incorporated in one and base in the other, inter‐catalysts interference was significantly diminished when introduces both into the CFM system (Figure 13d) [244]. Recent studies have integrated solid microspheres (SMs) with Pickering droplets in CFM systems for preventing coalesence‐induced efficiency losses. The closely packed SMs physically separate adjacent emulsion droplets facing where droplet‐droplet contact leads to merging over time [233]. Ti(Salen) complexes immobilized on SMs catalyze the initial step, while CALB within liquid droplets catalyzes the subsequent transformation, converting aldehydes with acetyl cyanide to chiral O‐acylated cyanohydrins (Figure 13e).

### 6.4 Double Emulsion Templated Microreactors

Double emulsion microreactors can address both the solvent affinity requirements of reactants and the spatial compartmentalization of catalysts [234]. A three‐phase microreactor system for addition reaction of stilbene and n‐bromosuccinimide which contains fluorocarbon oil (F‐oil), water, and hydrocarbon oil (H‐oil) was investigated [245]. The differential solubility of stilbene between F‐oil and H‐oil critically influences conversion rates, with H/F/W systems outperforming F/H/W system. Low stilbene solubility in F‐oil enables steady mass transfer from the innermost H‐oil phase through the F‐oil layer to the reaction interface (Figure 13f) [246]. Double emulsion templated‐enzyme compartmentalization system for cascade reaction was also studied [235, 247, 248, 249]. They fabricated the O/W emulsion where the ligand and molecular catalysts are in oil together. The emulsions are centrifugated to get a high intenal phase emulsion (HIPE) layer for using the highest surface area. And then, the HIPE in water with metal ion and excess oil was used for fabricating double emulsions.

Simultaneously, multi‐compartmental MOF cascade reactors was synthesized through coordination bonding between ligands in the inner and outer phases with metal ions in the middle phase. This approach achieves facile anchoring of enzyme and molecular catalyst in different layers for chemo‐enzymatic cascade reactions employing Grubbs catalyst and CALB (Figure 13g) [250]. They isolated the MOF particles and carried out the monophasic cascade reaction. The microgel system that can swell and shrink in a solvent and spontaneously adsorb at the oil‐water interface is also studied in double emulsion microreactors [236]. They prepared O/W/O double emulsions by the synergistic combination of hydrophobic microgels and hydrophilic lipase in twoimmiscible liquids. The concentration of polar additives could control the wettability of PNIPAM‐co‐4VP microgels, the double emulsion was spontaneously demulsified after the catalytic reaction. Subsequently, theproducts could be simply collected, and the double emul‐sions could be recycled multiple times for catalysis without the loss of enzymatic activity. Although there have been attempts to apply double emulsion microreactors to phase‐shifting reactions, most studies have focused on interior separation approaches, such as template‐based single‐phase cascade reactions or simultaneous independent reactions occurring in both phases, as described above [251, 252, 253, 254, 255]. Further research on phase‐shifting cascade reactions is therefore warranted.

### 6.5 Biomimetic Microcells based Cascade Microreactors

**FIGURE 14:**
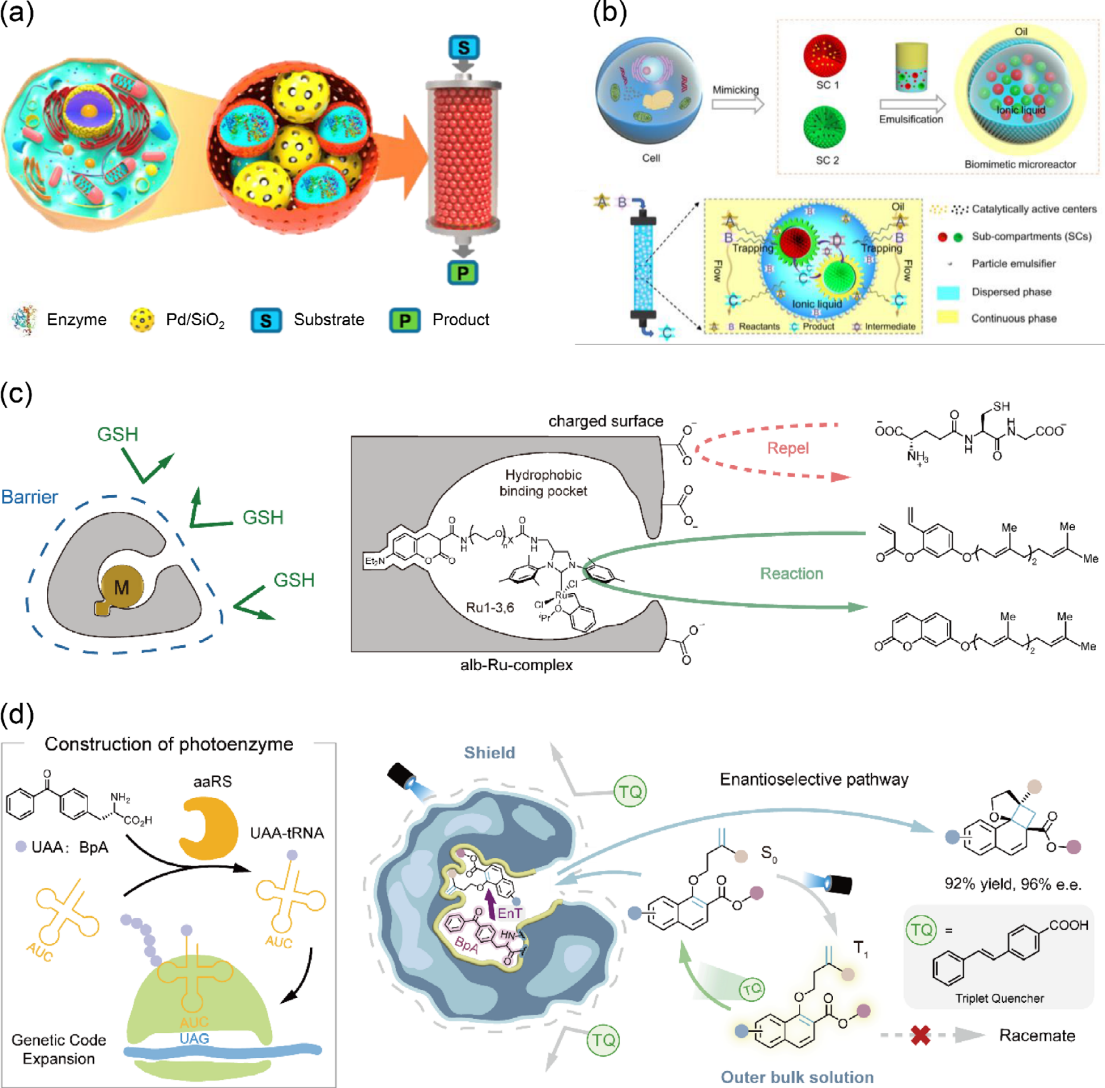
Advanced biomimetic microreactor systems. (a,b) Multi single emulsion‐templated microcells. Reproduced with permissions [253, 258]. Copyright 2023, American Chemical Society; Copyright 2022, Springer Nature. (c,d) Artificial enzymes. Reproduced with permissions [260, 261], Copyright 2025, Springer Nature; Copyright 2018, Springer Nature.

The comparmentalization of catalysts by constructing artifical wall between the phases enable the monophasic cascade reaction effectively, preventing the interference and aggregation between catalysts [182, 256]. Likewise, there are several studies about multi‐single emulsions templated biomimetic microcells [257]. One is combining stepwise Pickering emulsification with interface‐confined cross‐linking (Figure 14a) [258]. They co‐emulsified the palladium/silica nanocomposite with the crosslinked ionic liquid droplet to make multicompartmentalized liquid‐containing microreactors, whose interior architectures can be exquisitely tuned by regulating the number and size of subcompartments. They provided a bottom‐up route to construct biomimetic microcells for use in cascade catalysis. The other one is using two different types of mesoporous silica nanoparticles which loaded distinct catalyst in their holes [259]. They co‐emulsified in inner ionic liquid phase to construct a kind of “cell‐like” microreactor which is demonstrated to be capable of practical efficient cascade reactions. They regulated the amount of each catalyst ball and confirmed that the compartmentalization and enriching reactant properties of the microreactor were found to be crucial to the high catalysis efficiency (Figure 14b) [253].

### 6.6 Artificial Enzymes Based Microreactor

Artificial enzymes are rationally engineered to function as microreactors by integrating synthetic catalytic moieties into protein scaffold [262, 263]. They extended the concept of L–L interfacial catalysis by engineering a 3D protein‐confined pocket that can precisely control substrate binding and orientation. They mimic and even surpass the compartmentalized reactivity of L–L interfaces, enabling green catalysis with exceptional selectivity and efficiency under mild conditions. Artificial metalloenzymes (ArMs) are constructed by anchoring synthetic metal complexes within protein cavities. A seminal example is the development of a human serum albumin‐based ArM containing a ruthenium Hoveyda–Grubbs catalyst, designed for olefin metathesis in biological environments [264]. The deep hydrophobic binding pocket of albumin not only positions the substrate near the metal center but also crucially shields the ruthenium cofactor from deactivation by biological metabolites like glutathione (GSH) at physiologically relevant concentrations (up to 20 mM). This “protected activity” highlights the microreactor's dual function: enhancing catalysis through proximity effects while providing a biocompatible compartment that isolates the synthetic catalyst from hostile surroundings.

The concept extends to therapeutic applications, where glycosylated ArMs achieve targeted prodrug activation in cancer cells via ring‐closing metathesis, showcasing the potential for spatially controlled catalysis in complex biological settings (Figure 14c) [265]. The concept advances with artificial photoenzymes, where genetically encoded photosensitizers (e.g., 4‐benzoyl phenyl alanine, BpA) are installed within chiral protein cavities (Figure 14d) [260, 266]. The engineered pocket acts as a stereoselective micro‐interface, pre‐organizing substrates via multiple non‐covalent interactions before photoexcitation [261, 267]. This spatial arrangement is crucial for enantioselective triplet energy transfer, enabling previously elusive reactions like intramolecular [2+2]‐photocycloadditions with high enantioselectivity [268, 269]. Taking spatial control further, enzyme compartmentalization strategies intentionally segregate the catalytic cycle from the bulk solution to suppress competing pathways. By engineering the protein's surface charge and cavity access, tailored triplet quenchers can be excluded from the active site but remain active in the bulk phase. This creates a spatially resolved system where the desired enantioselective energy transfer occurs inside the protein microreactor, while background racemic reactions are quenched at the external “interface” with the solution [265]. This approach effectively solves a fundamental challenge in asymmetric photocatalysis and underscores the power of designing hierarchical micro‐environments to achieve orthogonal reaction control.

## 7 Conclusions and Future Perspectives

### 7.1 Key Insights: Interfacial Nanocatalyst Based Microreactors as Sustainable Platforms

In this review, we have comprehensively overviewed various types of L–L interfacial catalysis, ranging from conventional biphasic bulk systems to advanced continuous biphasic gel systems. We compared the respective advantages and limitations of each approach, with particular emphasis on PEMs, demonstrating their widespread applicability across diverse catalytic processes depending on the morphology and surface decoration strategies of PICs. By fragmenting two immiscible phases into small droplets, PEMs achieve significantly enhanced interfacial surface area and superior reactant selectivity, thereby delivering exceptional catalytic efficiency. Notably, we highlighted the substantial potential of PICs as green catalysts through their facile recovery and recyclability, further augmented by the incorporation of biodegradable polymers in catalyst design.

A critical advancement addressed in this review is the evolution beyond traditional PIC recovery methods, which conventionally required high‐energy physical desorption from the interface. We presented innovative strategies that enable straightforward catalyst recovery and recycling, significantly reducing energy consumption and operational complexity. Furthermore, we showcased the progression from simple PEMs to sophisticated cascade reaction microreactors employing Janus particles or multi‐compartment single emulsions. This strategic advancement transforms PEMs from limited reaction scope into versatile platforms capable of accommodating complex, multistep catalytic processes, thereby expanding their practical applicability in sustainable chemical synthesis.

### 7.2 Challenges in Industrial Translation and Future Perspectives

Despite the remarkable advantages demonstrated by rationally designed PICs which have superior catalytic efficiency, enhanced recyclability, and selective product recovery compared to conventional catalysts, several challenges remain for their industrial implementation [45, 59]. The primary obstacles are manufacturing cost and batch‐to‐batch homogeneity [44]. While PICs offer superior reusability, alternative catalysts with lower manufacturing costs often provide greater economic advantages despite their limited recyclability [270]. Moreover, from a thermodynamic perspective, achieving reaction rates in biphasic systems that substantially exceed those in homogeneous single‐phase reactions remains exceptionally challenging [72]. Nevertheless, for “phase‐shifting reactions” where the product phase differs from the reactant phase, PEMs represent a compelling solution by maximizing interfacial area between immiscible phases, thereby enhancing mass transfer efficiency. While PTCs currently dominates industrial applications for reactions where a new phase emerges from a single initial phase, we anticipate that introducing PEMs for phase‐shifting reactions could significantly enhance industrial competitiveness [271, 272, 273]. The facilitated mass transfer inherent to PEM systems could deliver reaction efficiencies surpassing those of conventional PTC approaches.

To realize this potential, simplification of PIC modification processes is imperative. Future research should prioritize bottom‐up synthetic strategies that maintain high batch consistency while streamlining surface functionalization procedures to reduce production costs. One of the most realistic solutions to these limitations is to replace conventional batch‐wise preparation with scale‐ready microfluidic or mesostructured continuous manufacturing. These platforms offer precise control over key structural and process parameters, including droplet size, interfacial particle coverage, catalyst loading, and residence time, which are essential for minimizing batch‐to‐batch variations and ensuring product consistency. Importantly, practical scale‐up may be better realized through numbering‐up, rather than by simply increasing reactor dimensions, as parallelization of identical production units can preserve the interfacial precision and reproducibility required for PIC fabrication. Accordingly, the integration of PIC synthesis with continuous manufacturing represents a promising pathway toward industrially relevant and standardized catalytic processes.

Additionally, machine learning‐assisted catalyst design can be promising strategy for identifying suitable catalysts. Two representative approaches can be highlighted: the first involves drastically narrowing the search space to reduce the experimental and developmental costs associated with catalyst screening, while the second employs simulations to evaluate catalytic efficiency in single‐atom alloy catalyst (SAAC) systems, thereby minimizing the use of expensive precious metals. A notable example of the former is that active learning was applied to the development of higher alcohol synthesis catalysts. By exploring compositional and reaction condition spaces, this approach compressed approximately 5 × 10^9^ possible combinations into just ≈86 experiments, reducing both the environmental footprint and associated costs by approximately 90% compared to conventional screening programs [274].

Regarding the reduction of catalyst raw material costs, one study demonstrated the power of DFT combined with AI‐driven data analysis to propose over 200 previously unreported promising SAAC [275]. In that work, they extremely diluted costly precious platinum to single‐atom level by dispersing onto host metals such as Cu, Ag, and Au, enabling a substantial reduction in noble metal loading. More recently, Bayesian optimization has been applied to investigate efficient iridium utilization, with a focus on identifying the optimal surface composition and chemical ordering of Ir‐TiO2 sytems [276]. Furthermore, there are efforts to discover high‐performance candidates entirely free of precious metals [277]. This study employed machine learning interatomic potentials to screen approximately 4000 candidate materials, leading to the identification of W‐NiFeOOH, which demonstrated remarkable stability over 5000 h at 2 V and 13.A cm^−^^2^. Taken together, these advances underscore that the integration of AI and catalysis represents a highly valuable approach for saving both cost and time in the context of future PIC fabrication and catalyst selection. Only through such cost‐effective and reproducible manufacturing approaches can PICs transition from promising laboratory‐scale systems to viable industrial catalytic platforms.

## Author Contributions

**B. Seo** and **J. Shin** contributed to conceptualization, data curation, methodology, writing – original draft; **M. Jang**, **K. Choi**, and **T. Pang** contributed to data curation and methodology; **F. Zhong** and **J. W. Kim** contributed to conceptualization, supervision, and writing – review and editing.

## Conflicts of Interest

The authors declare no conflicts of interest.

## Data Availability

The data that support the findings of this study are available from the corresponding author upon reasonable request.
